# Membrane lipid order predicts potency decline in therapeutic extracellular vesicles following handling, storage, and reconstitution stress

**DOI:** 10.3389/fbioe.2026.1795387

**Published:** 2026-04-13

**Authors:** Kenichi Tamura, Shinji Takeoka

**Affiliations:** 1 Cooperative Major in Advanced Biomedical Sciences of Tokyo Women’s Medical University and Waseda University, Tokyo, Japan; 2 Waseda Research Institute for Science and Engineering, Tokyo, Japan

**Keywords:** extracellular vesicles, generalized polarization, membrane lipid order, potency assay, quality control, stability

## Abstract

**Introduction:**

Potency assays for therapeutic extracellular vesicles (EVs) are widely recommended, yet common quality-control readouts (e.g., protein concentration and EV marker levels) are not always stability-indicating. We asked whether a membrane lipid order metric can sensitively detect potency deterioration of EVs after membrane damage and logistics-relevant handling, storage, and reconstitution stresses.

**Methods:**

Human fibroblast-derived EVs were isolated by ultracentrifugation. The study comprised two parts. First, as an artificial membrane-disruption model, EVs were exposed to graded concentrations of Triton X-100 to induce controlled membrane perturbation. Second, to mimic real-world post-manufacturing stresses, EVs were subjected to vortex mixing, −80 °C freeze–thaw cycling, or liquid nitrogen–assisted lyophilization followed by reconstitution in PBS. Membrane lipid order was quantified using a polarity-sensitive dye and expressed as corrected generalized polarization (cGP) on a standard plate reader. Conventional QC candidates (particle size by DLS, protein by BCA, and CD63 by ELISA) were evaluated in parallel. Potency was assessed as wound closure in an immortalized human keratinocyte scratch assay. Bivariate and multivariable regression analyses were performed to identify QC metric(s) that best predicted potency.

**Results:**

In the Triton model, EV potency declined even under ultralow detergent conditions that produced minimal changes in particle size, accompanied by increased extravesicular protein and a cGP shift consistent with reduced lipid order. This size–potency uncoupling and membrane-leakage signature motivated us to test whether the same lipid order readout could capture potency deterioration under practical stresses. In the real-world stress models, vortex mixing, freeze–thaw, and lyophilization-reconstitution again measurably decreased lipid order. Across stressed conditions within each EV lot, cGP tracked potency loss and showed stronger predictive performance than particle size, protein, or CD63. In contrast, baseline potency differences among independently manufactured lots were not well captured by cGP, supporting lipid order as a within-lot stability indicator rather than a between-lot potency ranking tool.

**Conclusion:**

A plate reader–based membrane lipid order metric provides a rapid, practical approach to detect potency decline of therapeutic EVs after membrane disruption and logistics-relevant stresses, and may support acceptance criteria for distribution control and bedside go/no-go decisions.

## 1 Introduction

Extracellular vesicles (EVs) are promising therapeutic modalities, and evidence suggests that EVs can recapitulate key pharmacological effects of their parent cells in regenerative medicine, including wound repair (Zhou et al., 2021; Meligy et al., 2025; Meriç et al., 2025). Alongside growing translational interest, recent guidance and position papers have emphasized EV characterization and quality control (Gimona et al., 2021; Welsh et al., 2024; Terai et al., 2025). Commonly implemented quality control (QC) attributes include particle size distribution, particle concentration, and bulk measurements such as protein and EV marker levels such as CD63 (Royo et al., 2020; Shekari et al., 2021). For therapeutic EVs, a key practical question is whether potency is preserved after storage, transport, and reconstitution. Several studies have shown that EV bioactivity can deteriorate during storage or freeze–thaw cycling even when particle size and bulk protein or marker measurements show little or no change, highlighting a central QC challenge for therapeutic EVs (Cheng et al., 2019; Levy et al., 2023). The lipid bilayer membrane is central to EV stability, uptake, and cargo protection (Zhuo et al., 2024). Lipid composition of EVs from multiple cell types has been characterized using lipidomics approaches such as LC/MS, and these studies have greatly advanced our understanding of EV membrane biology. However, lipidomics is not commonly implemented as a routine QC method, and—importantly—reports that use lipid composition measurements to predict therapeutic potency as a QC strategy remain limited. In practice, a small number of workflows include lipid-related readouts such as phosphate concentration as a surrogate of total phospholipid content, but such measurements do not inform membrane physical state (e.g., packing order or “membrane looseness”). This gap—limited access to simple QC tools that report membrane state—may contribute to why conventional QC attributes do not consistently reflect potency changes after storage-related handling. Membrane lipid order can be quantified by generalized polarization (GP) using dyes such as Laurdan and has been used primarily for biophysical characterization of liposomes and EVs. In contrast, its utility as a practical QC readout for predicting potency of therapeutic EVs remains unclear. Here, we evaluated a plate-reader-based membrane lipid order assay using LipiORDER.

**FIGURE 1:**
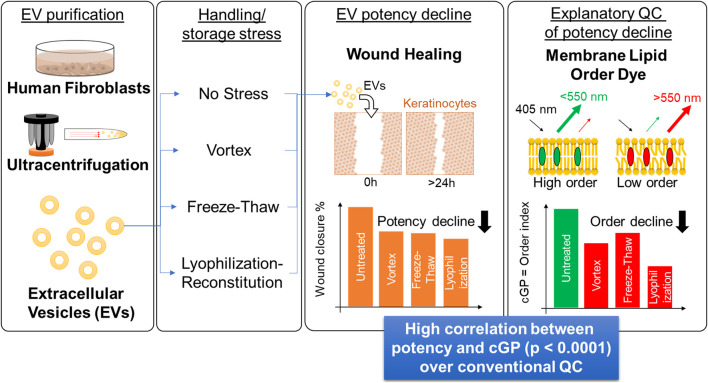
Graphical introduction to this research. In this study, we demonstrate that the membrane lipid order index, quantified as cGP, provides superior explanatory power as a QC metric than conventional QC readouts for accounting for the loss of EV wound-healing potency following typical handling and storage stresses.

This study has two parts. In the first part, we used Triton X-100 as a controlled membrane-disruption condition. Triton is commonly used to disrupt EV membranes in proteomics workflows and to test membrane dependence of EV-associated signals (Osteikoetxea et al., 2015; Bertokova et al., 2022). Here, we leveraged Triton to probe the relationship between membrane integrity, cargo retention, and functional potency. We report a notable phenomenon: under ultralow Triton conditions, potency decreased even when particle size appeared unchanged, and we observed increased extravesicular protein and reduced membrane lipid order. In the second part, we moved beyond this artificial disruption model and tested stresses more relevant to real-world handling and distribution—vortex mixing, −80 °C freeze–thaw cycling, and liquid nitrogen–assisted lyophilization followed by reconstitution. Using these stresses, we evaluated how membrane lipid order changes relate to potency changes and compared membrane lipid order with conventional QC candidates in their ability to predict potency decline. We show that membrane lipid order readout correlated the best among the tested candidates. The workflow of this important finding is summarized in Figure 1.

## 2 Materials and methods

### 2.1 EV preparation and purification

EVs were produced from human fibroblasts (TIG-120, JCRB Cell Bank, Osaka, Japan) and isolated by ultracentrifugation. Fibroblasts were maintained in DMEM (16971-55, Nacalai Tesque, Kyoto, Japan) supplemented with 10% v/v fetal bovine serum (S1810-500, biowest, Nuaillé, France), 5 ng/mL basic fibroblast growth factor (19155-36, Nacalai Tesque, Kyoto, Japan) and 1% v/v Penicillin/Streptomycin (168-23191, FUJIFILM Wako Pure Chemical Corp., Osaka, Japan). For EV collection, cells were seeded at 10,000 cells/cm^2^ and, after reaching confluence, the medium was replaced with serum-free DMEM. Conditioned medium was harvested after 72 h, clarified by filtration through a 0.22 μm filter, and ultracentrifuged at 35,000 rpm for 70 min using an Optima L-90K ultracentrifuge (Beckman Coulter, Brea, CA, United States). Ultracentrifugation was performed in 12 mL tubes. After the first ultracentrifugation, the supernatant was discarded, and the pellet was resuspended in 12 mL of phosphate-buffered saline (PBS; Thermo Fisher Scientific, MA, United States; osmolality ∼270–300 mOsm/kg, manufacturer specification). The suspension was then subjected to a second ultracentrifugation under the same conditions. After the second ultracentrifugation, the supernatant was discarded and the final EV pellet was resuspended in 1.0 mL PBS for downstream assays. Multiple independently manufactured EV lots were evaluated. Stress experiments were conducted using lots that had matched basal controls; additionally, independently manufactured basal lots were used for lot-to-lot comparisons.

### 2.2 Handling, storage, and reconstitution stress models

To generate controlled degrees of membrane perturbation, EVs were exposed to Triton X-100 (T8787-50 mL, Millipore sigma, Burlington, MA, United States) at graded concentrations (0.0001%, 0.001%, 0.01%, 0.1%, or 1% v/v). Triton-treated samples are denoted as “EV + Triton”, and concentration-specific conditions are labeled accordingly (e.g., EV + 0.0001% Triton).

To model practical post-manufacturing stresses, EVs were subjected to: (i) vortex mixing for 60 s, (ii) −80 °C freeze–thaw cycling, and (iii) liquid nitrogen–assisted lyophilization followed by reconstitution in PBS. Untreated EVs served as basal controls.

Vortex mixing was performed using Vortex-GENIE2 at 3,200 rpm for 60 s at room temperature (20 °C–25 °C). Vortex-treated samples are denoted as “EV + Vortex”.

For −80 °C freeze–thaw, EVs were aliquoted into 2 mL serum tubes (MS-4603B, Sumitomo Bakelite, Tokyo, Japan) and frozen at −80 °C for 4 h (single cycle). Samples were thawed by immersing half of the cryovial in a 37 °C water bath for 60 s. Freeze–thaw–treated samples are denoted as “EV + Freeze”.

For lyophilization, 1–3 mL of EV suspension was placed in a 10 mL round-bottom glass flask (TGK, Tokyo, Japan), immersed in liquid nitrogen for 60 s, and then mounted on a freeze dryer (DC401, Yamato Scientific, Tokyo, Japan). Samples were dried under <100 Pa for 4 h. Reconstitution was performed by adding PBS to the EV cake with a pipette followed by pipetting at least 10 times at room temperature (20 °C–25 °C). Lyophilized and reconstituted samples are denoted as “EV + Lyo”.

### 2.3 EV characterization by size, protein, and CD63

Hydrodynamic diameter distributions were measured by dynamic light scattering (DLS) using a Zetasizer (Malvern Panalytical, Malvern, UK), according to the manufacturer’s instructions. Because DLS is an intensity-weighted method and has known limitations when applied to heterogeneous and polydisperse populations, we report DLS as a standard, widely accepted sizing readout for EV preparations and interpret it in the context of these established limitations. Nevertheless, given its broad use and inclusion as an acceptable approach in MISEV 2023, DLS was adopted here to characterize EV size distributions and to support assessment of size consistency across conditions. Each condition was measured once (n = 1). For each sample, the instrument reports the hydrodynamic diameter and the polydispersity index (PDI) based on multiple short acquisitions averaged within a single run. Hereafter, “particle size” refers to the DLS-derived hydrodynamic diameter (Z-average), unless otherwise specified.

Protein concentration was quantified using a bicinchoninic acid (BCA) assay kit (Apro Science, Japan). The BCA working reagent was prepared by mixing reagents A and B at a 50:1 ratio. Standards and samples (25 μL each) were mixed with BCA working reagent (100 μL), incubated at 60 °C for 15 min, and then equilibrated to room temperature (20 °C–25 °C). Absorbance was measured at 562 nm using a Synergy H1 microplate reader (BioTek, Winooski, VT, United States).

CD63 concentration was quantified using a sandwich ELISA kit (Hakarel, Japan) according to the manufacturer’s instructions. Briefly, samples were captured on plates pre-coated with an anti-CD63 antibody, followed by incubation with an HRP-conjugated anti-CD63 detection antibody and substrate development. Absorbance was read on Synergy H1 reader at 450 nm.

### 2.4 BCA-based membrane integrity assay

Membrane integrity of EVs was assessed by two complementary approaches. First, after Triton, vortex, freeze–thaw, or lyophilization-reconstitution treatments, protein in the EV suspensions was assessed by BCA and expressed as a fold-change relative to the corresponding untreated EV suspension using the following Equation 1. This no-separation BCA readout reflects assay-accessible protein present in the EV preparation, which may include (i) soluble/extravesicular proteins, (ii) proteins associated with the EV exterior (e.g., a protein corona), (iii) membrane-associated proteins with surface-exposed domains, and (iv) intravesicular proteins that become accessible when membranes are loosened or compromised.

$$Normalized\;BCA\left(stress\right)=\frac{BCA\;of\;stress\;treated\;EV\;solution}{BCA\;of\;untreated\;EV\;solution}$$ (1)

Since the BCA assay is performed under strongly alkaline conditions (around pH ∼11), bicinchoninic acid is expected to be predominantly deprotonated (anionic) and therefore less likely to permeate the hydrophobic core of intact lipid bilayers. Accordingly, under unstressed conditions, intravesicular proteins are expected to be detected only to a limited extent, and increases in Equation 1 readout are most plausibly dominated by increases in extravesicular (and/or surface-associated) protein, with a possible minor contribution from membrane-compromise–dependent accessibility to intravesicular proteins. Accordingly, the readout should be interpreted as a measure of accessible protein rather than a strict compartment-specific quantification.

Second, after Triton exposure, vortex mixing, freeze–thaw, or lyophilization-reconstitution treatments, samples were processed using a 300-kDa molecular-weight-cutoff centrifugal filter (Vivaspin; Sartorius, Ann Arbor, MI, United States) to physically separate vesicle-sized material from soluble, extravesicular components. Samples were centrifuged at 6000 × *g* for 15 min, and protein in the flow-through was measured by BCA as an indicator of extravesicular protein isolated by the filtration step. Importantly, the flow-through signal reflects protein that is outside vesicles at the time of centrifugation, including (i) protein released to the extravesicular space by the applied stress itself and (ii) protein additionally released under the mechanical load of centrifugation from membranes that were loosened or compromised by the stress. The 300-kDa cutoff was used as a practical size-based separation between vesicle-sized material and soluble components; it does not imply that only <300-kDa cargo species leaked. To minimize the influence of lot-to-lot differences in starting protein content, the flow-through BCA readout was normalized in two steps: (i) flow-through BCA was divided by the corresponding pre-filtration suspension BCA for each condition using Equation 2, and (ii) this ratio was further normalized to the same ratio obtained for untreated EVs using Equation 3.

$$Normalized\;BCA\;\left(centrifuge\right)=\frac{BCA\;of\;flowthrough\;of\;EV\;solution}{BCA\;of\;EV\;solution}$$ (2)

$$Normalized\;BCA\;\left(stress+centrifuge\right)=\frac{Normalized\;BCA\;\;\left(centrifuge\right)\;of\;stress\;treated\;EV\;solution}{Normalized\;BCA\;\left(centrifuge\right)\;of\;untreated\;EV\;solution}$$ (3)

### 2.5 Membrane lipid order measurement

EV samples or PBS controls were incubated with LipiORDER (FDV-0041, Funakoshi, Japan), a polarity-sensitive membrane lipid order dye. A 1 mM LipiORDER stock solution was prepared in dimethyl sulfoxide (DMSO) and stored at −20 °C. The dye stock was added at 0.1% v/v to EV suspensions or PBS to a final concentration of 1 μM and incubated at 37 °C for 10 min. Fluorescence emission spectra (450–700 nm) were acquired with 405 nm excitation using a Synergy H1 microplate reader (BioTek) at 20 °C.

Membrane lipid order was expressed as corrected generalized polarization (GP), a widely used metric for quantifying membrane lipid order with polarity-sensitive probes such as Laurdan and related dyes (Parolini et al., 2009; Suga et al., 2021; Yasuda et al., 2022; Auquière et al., 2025; Peruzzi et al., 2024; Seegobin et al., 2025). GP-based approaches have also been applied in prior EV studies to assess membrane lipid order/fluidity. Importantly, because GP is computed from a ratio of two emission channels, prior work emphasizes that instrumental channel imbalance can bias raw GP values and therefore introduces a calibration/correction factor (often termed a “G factor”) derived from a reference measurement to obtain corrected GP values (Brewer et al., 2010). In this study, to reduce plate-to-plate variability and measurement-day effects in a plate-reader setting, we used a corrected GP (cGP) in which a plate-specific correction factor was derived from PBS measurements on the same plate. Our cGP is a practical plate-reader adaptation of the established corrected-GP concept. cGP was calculated from the following Equation 4 using fluorescence intensities at 540 nm and 580 nm.

$$cGP=\frac{EV\;I\;\left(540\;nm\right)-k\times EV\;I\;\left(580\;nm\right)}{EV\;I\;\left(540\;nm\right)+k\times EV\;I\;\left(580\;nm\right)}$$ (4)

Here, “I” denotes the normalized fluorescence emission intensity (arbitrary units) recorded at the specified emission wavelength. For each EV sample, “EV I (540 nm)” and “EV I (580 nm)” represent the emission intensities measured from the EV-containing well at the short-wavelength channel (540 nm) and the long-wavelength channel (580 nm), respectively. In parallel, PBS was measured on the same plate as a blank control. “PBS I (540 nm)” and “PBS I (580 nm)” represent the corresponding emission intensities measured from PBS-only wells at the same wavelengths, reflecting plate- and instrument-specific spectral bias and background under the same settings. A plate-specific correction factor (k) was calculated from PBS blanks on the same plate using the following Equation 5.

$$k=\frac{PBS\;I\;\left(540\;nm\right)}{PBS\;I\;\left(580\;nm\right)}$$ (5)

A ΔcGP was defined relative to the basal/control condition for each EV lot, calculated as Equation 6.

$$\Delta cGP=cGP\;\left(stress\;treated\right)-cGP\;\left(untreated\right)$$ (6)

ΔcGP <0 indicates reduced membrane lipid order (loosening of lipid packing in bilayer membrane). In line with the manufacturer’s guidance, the two emission channels were selected on opposite sides of 550 nm.

### 2.6 Keratinocyte scratch (wound closure) assay for potency

Scratch assays were performed using immortalized human keratinocytes (PHK16-0b, JCRB Cell Bank, Osaka, Japan). Cells were maintained in KGM-Gold (Lonza, Basel, Switzerland). For the assay, cells were seeded in 48-well plates at 1 × 10^6^ cells/mL and cultured to reach a confluent monolayer by the next day.

Scratches were created using a P1000 pipette tip. Wells were rinsed three times with KBM-Gold, which is KGM-Gold basal medium without supplements, then cells were incubated with an in-house made assay medium based on KGM-Gold in which factors primarily related to keratinocyte migration and proliferation were omitted. Test samples (EV preparations, PBS vehicle, or EGF) were added to the assay medium at 10% v/v. Notably, EV dosing in the wound-healing assay was not normalized to protein concentration or CD63 amount, because these attributes were evaluated as candidate predictors of potency. Instead, dosing was controlled by standardizing the EV production workflow (cell seeding density, conditioned-medium collection volume, and final PBS resuspension volume) and adding an equal volume fraction of the resulting EV suspension to each well (10% v/v). Images were acquired at fixed positions at 0 h and 24 h, or at 0 h and 72 h depending on the experiment, and wound area was quantified using ImageJ. For Triton X-100–exposed EV experiments, wound closure was analyzed at 24 h because Triton-related effects on keratinocytes were observed to become pronounced between 24 and 48 h (data not shown). For all other conditions (vortex mixing, freeze–thaw, and lyophilization-reconstitution), wound closure was analyzed at 72 h. Potency was expressed as percent wound closure using the following Equation 7.

$$Wound\;Closure\;\%=\left\{1-\frac{Wound\;Area\;\left(t\right)}{Wound\;Area\;\left(0\right)}\right\}\times 100$$ (7)

Wound Area (0) is the wound area at 0 h and Wound Area (t) is the wound area at the indicated time point (24 h or 72 h). A value of 100% indicates complete closure of the scratch (no remaining wound gap), 0% indicates no closure from baseline (wound area unchanged from 0 h), and 50% indicates partial closure with the wound area reduced to half of the initial area.

To normalize wound closure to the 0% Triton condition, we divided the wound closure percentage measured in samples exposed to 0.001% (or 0.0001%) Triton X-100 by the wound closure percentage measured in the 0% Triton condition using the following Equation 8.

$$0\%Triton\;normalized\;wound\;closure\;\%=\frac{Wound\;Closure\;\%\;of\;sample\;with\;0.001\%\;or\;0.0001\%Triton}{Wound\;Closure\;\%\;of\;sample\;with\;0\%Triton}$$ (8)

Unless otherwise specified, each experimental condition was evaluated in duplicate wells (technical replicates). EV-related experiments were repeated using multiple independently manufactured EV lots (typically three lots).

### 2.7 Regression modeling of potency *versus* QC candidates

Associations between potency and candidate predictors (size metrics, protein, CD63, and membrane lipid order indices) were evaluated by linear regression. We first performed bivariate analyses to describe unadjusted relationships between each QC candidate and scratch-assay potency. We then performed multivariable regression to assess whether each candidate remained associated with potency after adjustment for the other measured parameters (i.e., independent contribution). Statistical significance of coefficients was assessed using Student’s t-test, with p values reported. For correlation analyses between wound-closure potency and QC candidates, we used six independently manufactured EV lots, labeled EV01–EV06. The numbering has no biological meaning and simply reflects the order of preparation; lots were intentionally analyzed separately to enable assessment of both within-lot stress effects and between-lot variability under basal conditions. As an exploratory particle-count proxy, we calculated an “equivalent particle-count proxy (arbitrary units)” from the LipiORDER fluorescence area-under-the-curve (AUC; lipid-volume proxy) and the DLS-derived particle size assuming spherical particles. Specifically, the particle size was converted to a radius (*r* = d/2), and the AUC was normalized by the corresponding spherical surface area (4πr^2^) to obtain a surface area–normalized proxy. This metric is not intended as an absolute particle concentration and is not part of MISEV recommendations but was used as a sensitivity analysis to assess whether conclusions were robust to a particle-count–like normalization. Stratified analyses were performed within EV lots (including stressed conditions) and across lots under basal conditions. Analyses were performed in JMP (SAS Institute).

## 3 Results

### 3.1 Ultralow detergent exposure reveals potency loss without major size changes

**FIGURE 2:**
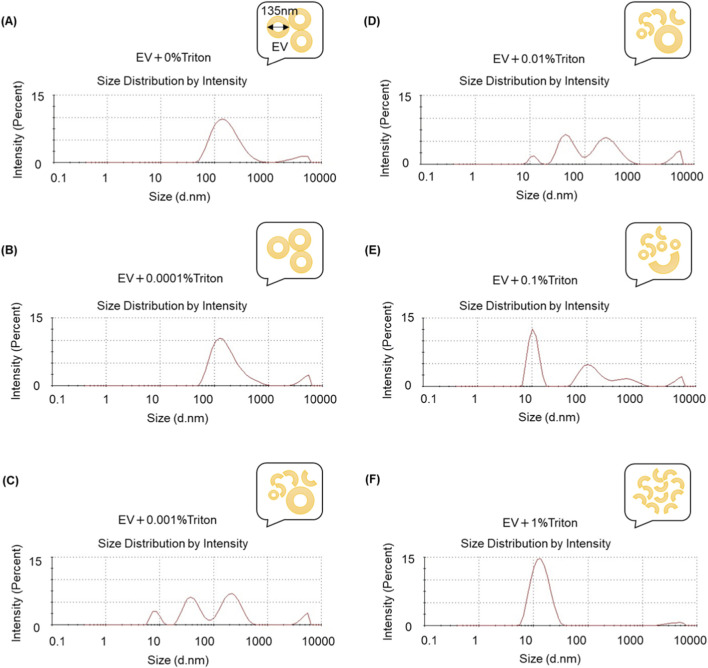
EV size distributions measured by DLS after graded Triton X-100 exposure. Representative intensity-weighted DLS size distributions of fibroblast derived EVs following exposure to graded Triton X-100 concentrations (v/v): **(A)** 0%, **(B)** 0.0001%, **(C)** 0.001%, **(D)** 0.01%, **(E)** 0.1%, **(F)** 1%. Insets show schematic cartoons summarizing the observed DLS distribution patterns for each condition. n = 1. Each DLS measurement comprises multiple short acquisitions that are averaged; replicate measurements were limited by the ∼1 mL sample volume required per run.

EVs were isolated from human fibroblast conditioned medium by ultracentrifugation; the source-cell morphology under EV production conditions and enrichment of the EV marker CD63 in the purified EV fraction are shown in Supplementary Figure S1. Basal EVs exhibited a predominant size population around 135 nm by DLS (Figure 2). To establish a controlled reference condition for sub-lytic membrane perturbation, EVs were then exposed to graded concentrations of Triton X-100. Low Triton concentrations (EV + 0.0001% Triton and EV + 0.001% Triton) preserved a dominant peak around 135 nm, whereas higher concentrations (EV + 0.01% Trion, EV + 0.1% Triton, and EV + 1% Triton) shifted the dominant peak toward smaller apparent diameters with increased heterogeneity. To aid interpretation, schematic cartoons summarizing the observed size-distribution patterns are shown as insets in the upper-right corner of each DLS plot, illustrating progressively greater membrane disruption with increasing Triton X-100 concentration. We did not systematically perform serial dilution to test dilution-dependence of the large-diameter tail; thus, we interpret this signal as consistent with aggregation but acknowledge that DLS readouts can be influenced by polydispersity and scattering bias. In addition, gentle pipetting was used to avoid additional membrane perturbation before measurement, which may have been insufficient to disperse loosely associated aggregates and could have contributed to the observed clumping signal. Notably, the apparent large-diameter tail was reduced at higher Triton concentrations, which is consistent with this component representing aggregation/clumping that can be dispersed or eliminated under stronger detergent conditions.

**FIGURE 3:**
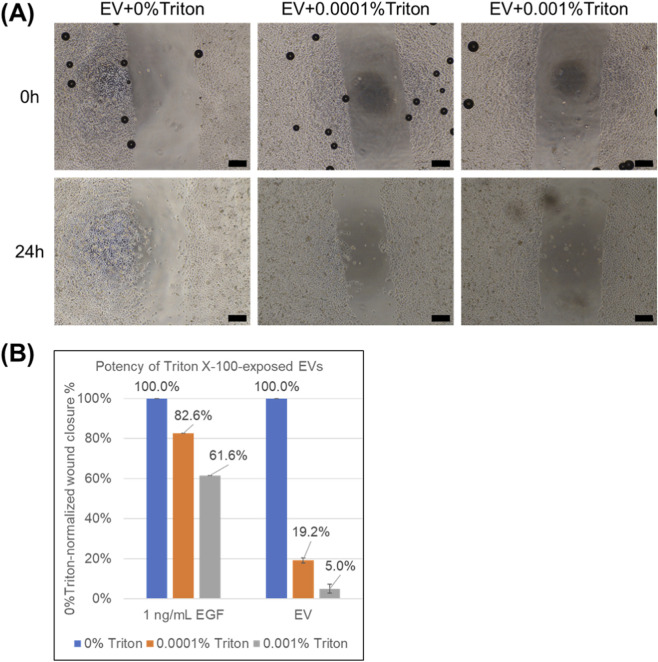
Scratch-assay wound-closure readout after ultralow Triton X-100 exposure. **(A)** Representative phase-contrast images (4×) of immortalized human keratinocyte scratch assay using EV preparations exposed to Triton X-100 at 0%, 0.0001%, or 0.001% v/v are shown. Scale bar: 200 µm. **(B)** 0%Triton-normalized wound closure (%) are shown. Bars show mean ± SD (EVs: n = 3 independent lots, each assayed in duplicate wells; EGF: duplicate wells in one experiment).

**FIGURE 4:**
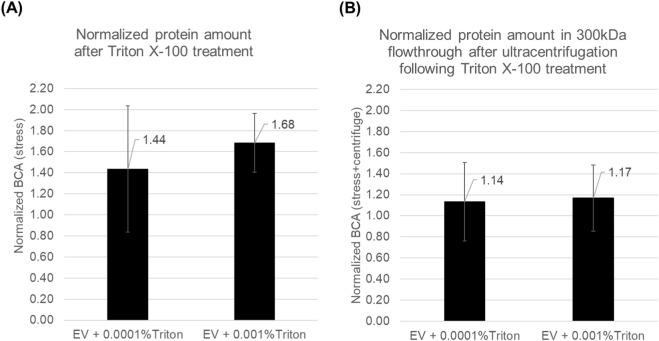
BCA-detected protein in EV suspensions and in the 300-kDa flow-through after ultralow Triton X-100 exposure. **(A)** Normalized BCA (stress) for EV + 0.0001% Triton and EV + 0.001% Triton are shown. **(B)** Normalized BCA (stress + centrifuge) for EV + 0.0001% Triton and EV + 0.001% Triton are shown. Data are mean ± SD (n = 3 independent lots).

Despite minimal size changes at 0.0001% Triton X-100, the scratch assay revealed a marked potency loss, with an ∼80% reduction in wound-closure stimulation at 24 h compared with EV+0%Triton (Figure 3). Three independent EV production lots were tested in this experiment. As shown in Supplementary Figure S2, Triton-only vehicle conditions at 0%, 0.0001%, and 0.001% showed at most a mild reduction in scratch closure rate, which was substantially smaller than the loss observed for EVs exposed to ultralow Triton. Thus, while a modest Triton effect cannot be completely excluded, the magnitude of the potency decrease in the EV + Triton conditions is unlikely to be explained by vehicle alone under these low-detergent conditions. Together, these findings show that EV potency can deteriorate substantially even when particle size distributions appear preserved, highlighting a key limitation of size-based QC for detecting stability-relevant changes. This observation motivated us to test whether protein detectability/leakage changes after Triton exposure could provide an additional clue to the underlying membrane-level alteration (Figure 4).

### 3.2 Ultralow detergent increases extravesicular protein

Detergent exposure increased protein detected by BCA (Figure 4). As ultralow Triton X-100 caused a marked loss of scratch-assay potency despite minimal changes in particle size (Figures 2, 3), we hypothesized that detergent treatment induced sub-lytic membrane compromise and increased the amount of protein detectable outside vesicles (e.g., by leakage and/or increased accessibility of vesicle-protected proteins). We therefore quantified BCA-detectable protein in the post-treatment EV suspensions.

In Figure 4A, even ultralow Triton X-100 increased protein detected in the EV suspension despite minimal changes in particle size, shown as a fold-change relative to the pre-exposure condition. However, given the variability observed across replicates and the limited sample size (n = 3), we describe these changes as trends rather than definitive between-group differences. This BCA readout could, in principle, reflect not only proteins already present outside vesicles but also proteins that become accessible when membranes are loosened. However, the BCA assay is performed under strongly alkaline conditions, under which bicinchoninic acid is expected to be predominantly deprotonated (anionic) and therefore less likely to permeate the hydrophobic core of intact lipid bilayers; thus, the signal is most plausibly dominated by protein present outside vesicles or on vesicle surfaces.

Figure 4B assesses leakage under an added mechanical load and is expected to more selectively represent extravesicular protein. After Triton exposure, samples were subjected to 300-kDa cutoff centrifugal filtration. Across concentrations, Triton increased the amount of protein detected in the flow-through, indicating an increased propensity for protein to be present outside vesicles. Because the filtration step applies centrifugal stress to the sample, these data further suggest that detergent exposure renders EV membranes more susceptible to cargo release under mechanical loading, revealing additional leaked protein beyond what is apparent without centrifugation.

### 3.3 Membrane lipid order is decreased by ultra-low detergent and predicts potency decline

**FIGURE 5:**
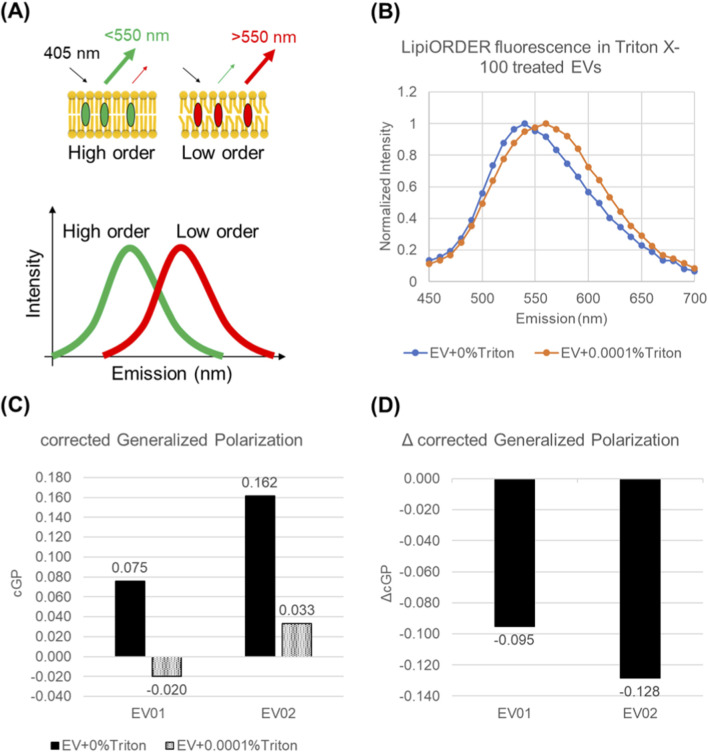
LipiORDER fluorescence spectra and GP-derived membrane-order metrics after Triton X-100 exposure. **(A)** Schematic of the LipiORDER emission spectra. **(B)** Normalized LipiORDER emission spectra (excitation 405 nm) of a representative untreated EVs (0% Triton) and ultralow Triton-treated EVs (0.0001% Triton) from lot EV02. Data are from triplicate wells in one experiment. **(C)** Corrected generalized polarization (cGP) calculated from two emission channels on opposite sides of 550 nm (540 nm and 580 nm) measured in EV01 and EV02. Data are from triplicate wells for each lot. **(D)** Stress-induced change in membrane lipid order expressed as ΔcGP.

Following the observation that ultralow Triton X-100 markedly reduced scratch-assay potency and increased extravesicular protein despite minimal changes in particle size (Figures 2–4), we next sought a simpler and more sensitive readout to quantify membrane-level damage. We therefore tested a polarity-sensitive membrane lipid order dye (LipiORDER) and found a clear spectral shift in its fluorescence after Triton exposure (Figure 5): the emission peak shifted from 540 nm to 560 nm, consistent with reduced membrane lipid order according to the manufacturer’s guidance. To quantify this peak shift, we calculated corrected generalized polarization (cGP) using two emission channels on opposite sides of 550 nm (540 nm and 580 nm). Across both EV lots tested, Triton exposure shifted cGP toward more negative values compared with untreated EVs, indicating membrane loosening. The stress-induced change was summarized as ΔcGP, supporting cGP as a quantitative index of EV membrane lipid order.

In addition, because Triton is a surfactant, we evaluated Triton-only (no EV) LipiORDER spectra and observed an emission peak above 550 nm, consistent with LipiORDER partitioning into detergent micelles, which likely represent a low-order environment (Supplementary Figure S3). In our EV experiments, Triton was added to the EV suspension first, allowing Triton–membrane interactions to occur before the subsequent addition of LipiORDER; therefore, it is unlikely that LipiORDER would preferentially partition into Triton micelles in the presence of abundant EV membranes. Nevertheless, a fraction of unbound (“free”) Triton is expected to remain in solution, and we cannot fully exclude a minor contribution of micelle-associated LipiORDER signal arising from this free-detergent fraction, particularly at ultralow Triton concentrations. Accordingly, we interpret the Triton experiment as a controlled perturbation reference that motivated the subsequent real-world stress analyses, which do not involve detergents. With these considerations in mind, having established this membrane lipid order readout under a controlled detergent perturbation, we next tested whether it can capture membrane loosening induced by practical, real-world stresses relevant to EV handling, storage, and reconstitution (vortex mixing, freeze–thaw, and lyophilization-reconstitution), as described in the following sections.

### 3.4 Practical handling/storage stresses cause modest, stress-dependent changes in BCA-detected protein

**FIGURE 6:**
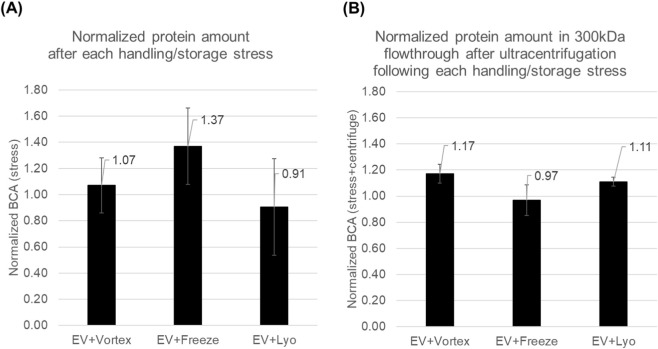
BCA-detectable protein after practical handling/storage stresses with and without centrifugal filtration. **(A)** Normalized BCA (stress) for EV + Vortex, EV + Freeze, and EV + Lyo are shown. **(B)** Normalized BCA (stress + centrifuge) for EV + Vortex, EV + Freeze, and EV + Lyo are shown. Data are mean ± SD (n = 3 independent lots).

Practical stresses also altered the amount of protein detected by BCA, but the magnitude of change was modest and the patterns differed by stress type (Figure 6). In Figure 6A, BCA values are shown as fold-changes normalized to untreated EVs set to 1.0 and represent protein that becomes detectable in the suspension after the stress alone (vortex mixing, freeze–thaw, or lyophilization-reconstitution). As in the detergent experiments, this BCA readout without centrifugation reflects protein accessible to the assay. It is most plausibly dominated by proteins already present outside vesicles or on vesicle surfaces, although—as discussed above—membrane loosening could also increase accessibility of luminal proteins without requiring complete vesicle disruption. Notably, the direction of change was not uniform (e.g., lyophilization was <1.0 in the direct readout), suggesting appreciable measurement variability in this assay; accordingly, smaller deviations from 1.0 (e.g., ∼1.07) should be interpreted cautiously and were not as prominent as the detergent-driven shifts.

Figure 6B evaluates protein measured in the 300-kDa flow-through after centrifugal filtration and is expected to more selectively reflect extravesicular protein, while also incorporating the combined effects of the applied stress and centrifugal loading on cargo release. Here, freeze–thaw showed no further increase after filtration (0.97-fold), whereas vortex mixing showed a modest upward trend (∼1.17-fold) and lyophilization a smaller change (∼1.11-fold). Overall, these practical-stress conditions did not produce the pronounced increases seen with Triton; however, because some conditions (notably freeze–thaw in the direct readout and vortex mixing in the filtration readout) suggested a tendency toward increased detectable/extravesicular protein, we next evaluated membrane lipid order as a more sensitive and practical readout of membrane-level perturbation under these real-world stresses.

### 3.5 Membrane lipid order decreased after logistics-relevant handling/storage stresses

**FIGURE 7:**
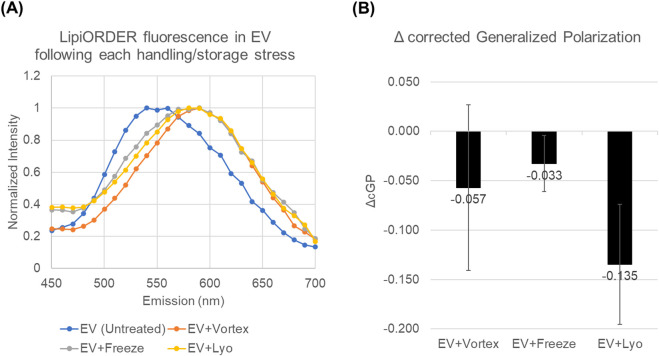
LipiORDER spectra and ΔcGP after practical handling/storage stresses. **(A)** Representative normalized LipiORDER emission spectra (excitation 405 nm) for untreated EVs and stressed EVs from lot EV01. **(B)** Stress-induced change in membrane lipid order expressed as ΔcGP. Data are shown as mean ± SD (n = 3 independent lots for EV + Vortex and EV + Freeze; n = 5 independent lots for EV + Lyo).

LipiORDER fluorescence spectra of EVs showed stress-dependent changes in emission profiles after vortex mixing, freeze–thaw, and lyophilization-reconstitution (Figure 7A). Relative to untreated EVs, stressed samples exhibited a modest shift of the spectral distribution toward longer wavelengths (i.e., increased signal on the >550 nm side), consistent with reduced membrane lipid packing order. Specifically, the emission peak maxima were 540 nm for untreated EVs, 590 nm for vortex- and freeze-thaw–treated EVs, and 580 nm for lyophilization-reconstitution–treated EVs. To quantify these spectral changes in a plate-reader–compatible manner, we calculated corrected generalized polarization (cGP) using two emission channels on opposite sides of 550 nm and summarized the stress effect as ΔcGP (Figure 7B). Notably, whereas BCA-based protein readouts showed only modest or variable changes depending on the stress condition, the membrane lipid order metric provided a clearer and more direct indication of stress-associated membrane loosening. All three stresses shifted ΔcGP in the negative direction, with lyophilization-reconstitution showing the largest decrease (ΔcGP = -0.135), followed by freeze-thaw (ΔcGP = -0.033) and vortex mixing (ΔcGP = -0.057). The control (untreated EV) is defined as the reference at ΔcGP = 0, and stressed groups are plotted as deviations from this control. Notably, the EV + vortex condition included 0 within its confidence interval for ΔcGP, whereas the freeze-thaw and lyophilization-reconstitution conditions did not. This suggests that vortex-induced membrane-order changes were smaller and/or more variable in our dataset, and should be interpreted as a modest trend rather than a robust shift. These results indicate that common handling/storage stresses can loosen EV membranes, motivating subsequent analyses of how this membrane lipid order readout relates to functional potency across conditions.

### 3.6 Membrane lipid order predicts potency decline across stressed conditions

**FIGURE 8:**
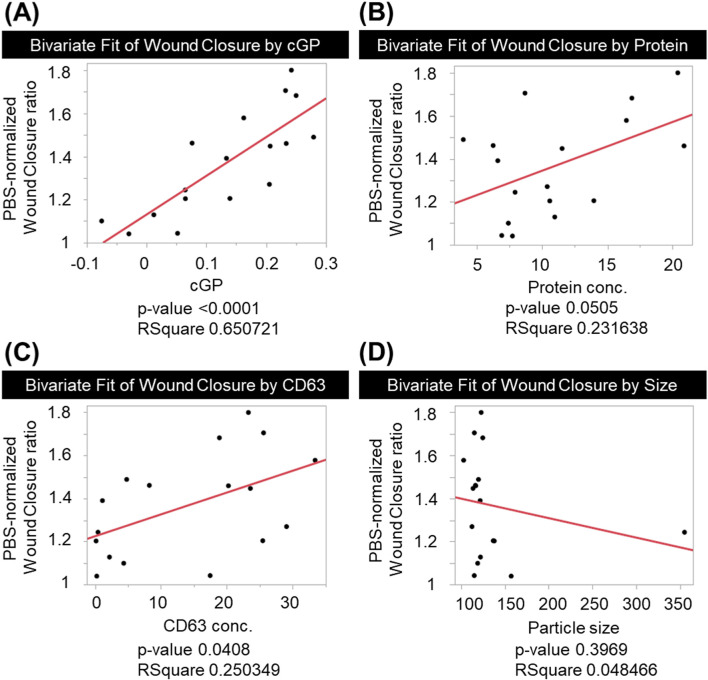
Bivariate relationships between keratinocyte scratch-assay potency (y-axis) and candidate QC measures (x-axes) across stressed EV samples. Scratch-assay potency is expressed as the ratio of wound-closure (%) relative to the matched PBS vehicle control in the same experiment. Predictor variables are **(A)** cGP, **(B)** protein (µg/mL), **(C)** CD63 (ng/mL), and **(D)** particle size (nm). Red lines indicate fitted linear regression trends for each bivariate model.

Having established cGP as an EV membrane lipid order index, we next examined its relationship with potency and compared it with conventional QC candidates, including particle size, protein, and CD63 using bivariate analysis. Potency was quantified as wound-closure (%) in the keratinocyte scratch assay. To control for day-to-day variability in baseline closure, wound-closure values for each EV condition were expressed relative to the matched PBS vehicle control measured in the same experiment (i.e., potency was analyzed as an EV-to-PBS ratio of wound closure). Across stressed EV samples, the membrane lipid order metric showed the strongest association with scratch-assay potency (p < 0.0001), whereas protein (p = 0.0505) and CD63 (p = 0.0408) showed weaker associations and particle size was not informative (p = 0.3969) (Figure 8). The directionality indicated that greater membrane loosening corresponded to lower potency. Because an outlier in the particle-size analysis could influence the regression slope (Figure 8D), we performed a sensitivity analysis excluding this point while retaining it in the scatter plot for transparency. The primary conclusion—that cGP showed the strongest association with potency—was unchanged; however, after excluding the outlier, particle size showed a tighter, directionally consistent trend, with smaller diameters tending to be associated with higher potency (Supplementary Figure S4D).

We also performed a least-squares multiple linear regression with scratch-assay potency as the dependent variable and cGP, protein, CD63, and particle size as candidate predictors (n = 17). The overall model was statistically significant (F (4,12) = 6.2329, p = 0.0059). Model fit statistics were RSquare = 0.675076, adjusted RSquare = 0.566768, RMSE = 0.156579, and the mean of the dependent variable was 1.368927. Regression coefficients (estimate and p value) indicated that only cGP remained statistically significant after adjustment for the other variables (β = 1.7053911, p = 0.0031). In contrast, protein (p = 0.3691), CD63 (p = 0.7348), and particle size (p = 0.8546) were not significant in the multivariable model. All the quantitative data used in this correlation analysis is listed in Table 1.

**TABLE 1:** List of underlying measurements used in the bivariate and multivariable analyses of scratch-assay potency *versus* QC candidates.

EV lot #	Stress condition	PBS-normalized wound closure ratio	Protein conc. (µg/mL)	CD63 conc. (ng/mL)	Particle size (nm) (PDI)	cGP	Equivalent particle-count proxy
EV01	Untreated	1.46	6.23	8.21	116.1 (0.2228)	0.075	0.170
	Vortex	1.10	7.39	4.32	118.7 (0.2840)	−0.076	0.052
	Freeze-thaw	1.13	10.95	2.16	121.9 (0.3189)	0.011	0.484
	Lyophilization-reconstitution	1.04	7.71	0.23	156.9 (0.2550)	−0.030	0.194
EV02	Untreated	1.58	16.44	33.37	102.7 (0.2619)	0.162	0.064
EV03	Untreated	1.71	8.67	25.55	114.9 (0.2658)	0.232	0.124
	Vortex	1.27	10.38	29.03	112.2 (0.2751)	0.205	0.298
	Freeze-thaw	1.45	11.51	23.54	113.4 (0.2637)	0.206	0.010
	Lyophilization-reconstitution	1.04	6.89	17.44	114.8 (0.2861)	0.051	1.312
EV04	Untreated	1.80	20.38	23.24	122.7 (0.2358)	0.242	1.304
	Vortex	1.68	16.86	18.84	124.5 (0.2303)	0.250	1.064
	Freeze-thaw	1.46	20.86	20.21	116.5 (0.2139)	0.233	0.329
	Lyophilization-reconstitution	1.21	13.95	25.42	136.5 (0.4476)	0.139	1.038
EV05	Untreated	1.49	3.94	4.77	119.6 (0.1945)	0.279	1.631
	Lyophilization-reconstitution	1.25	7.91	0.42	354.5 (0.4971)	0.064	1.562
EV06	Untreated	1.39	6.58	1.09	121.5 (0.1801)	0.133	0.490
	Lyophilization-reconstitution	1.20	10.57	0.13	137.6 (0.2510)	0.064	1.662

### 3.7 Stratified analysis 1: membrane lipid order is not sensitive for baseline between-lot potency

Among independently manufactured basal (non-stressed) lots, cGP did not explain lot-to-lot potency differences (Supplementary Figure S5; p = 0.2603). In contrast, protein and CD63 showed trend-level associations, whereas particle size was not informative (Supplementary Figure S5; protein p = 0.0922, CD63 p = 0.0941, particle size p = 0.999). These results suggest that baseline potency differences across EV lots are likely driven by lot-specific differences in EV composition/cargo and by QC attributes that reflect EV abundance and/or purity (e.g., protein and CD63), and are therefore not readily explained by membrane lipid order alone.

### 3.8 Stratified analysis 2: across stress types, membrane lipid order shows the most robust association with potency

Stratified analyses by stress types showed that cGP consistently tracked potency changes induced by each stress (Supplementary Figures S6–S8). To examine whether the potency-predicting QC metric depends on the type of logistics-relevant stress, we performed stress-stratified bivariate regressions between scratch-assay potency (PBS-normalized wound closure ratio) and each QC candidate (cGP, protein, CD63, and particle size) using three independently manufactured EV lots per stress condition. Across all three stresses, cGP showed the strongest positive association with potency, indicating that greater membrane lipid order was linked to higher wound-closure activity and that membrane loosening was linked to potency decline.

For vortex mixing, cGP showed the strongest relationship with potency (p = 0.0569; RSquare = 0.637, corresponding to a high correlation magnitude), whereas protein (p = 0.1544), CD63 (p = 0.3317), and particle size (p = 0.3428) were not significant (Supplementary Figure S6). For freeze–thaw, cGP again showed the strongest association (p = 0.0561; RSquare = 0.640), while protein and particle size were clearly non-informative (p = 0.5731 and p = 0.9003, respectively). CD63 showed a stress-dependent borderline association in this subgroup (p = 0.0576), but this trend was not consistently observed across other stresses (Supplementary Figure S7). For lyophilization-reconstitution, cGP remained the most predictive metric and was statistically significant (p = 0.0033; RSquare = 0.680), whereas protein (p = 0.2940), CD63 (p = 0.2149), and particle size (p = 0.4823) showed weaker or no associations (Supplementary Figure S8).

Together, these stratified results indicate that membrane lipid order (cGP) is the only QC candidate that reproducibly shows a strong, positive correlation with potency across distinct stress modalities, supporting its utility as a stability-indicating metric under practical handling and storage conditions.

### 3.9 Stratified analysis 3: cGP shows consistent directionality and higher fit for within-lot potency decline

Within individual EV lots that included stress-degraded samples, the relationship between membrane lipid order (cGP) and scratch-assay potency was directionally consistent across lots, showing positive regression coefficients in every evaluable lot (Supplementary Figures S9–S13). In contrast, protein, CD63, and particle size exhibited lot-dependent directions (positive or negative slopes), indicating less consistent within-lot tracking of potency decline.

To derive these within-lot estimates, we assessed correlations between PBS-normalized wound-closure potency and multiple QC candidates (cGP, protein, CD63, and particle size) separately for each EV lot. Because EV02 was evaluated only in the untreated (initial) state, within-lot correlations could not be estimated for that lot; therefore, the stratified analysis included EV01 and EV03–EV06. Across these lots, cGP not only showed consistent directionality but also tended to yield larger goodness-of-fit (RSquare) than conventional QC candidates, supporting membrane lipid order as a stability-indicating attribute for detecting within-lot potency deterioration.

### 3.10 Exploratory analysis using an equivalent particle-count proxy

As an additional sensitivity analysis addressing the lack of direct particle-count instrumentation (e.g., NTA/TRPS), we derived an “equivalent particle-count proxy (arbitrary units)” from the LipiORDER fluorescence area-under-the-curve (AUC; lipid-amount proxy) and the DLS-derived particle size under a spherical-particle assumption. Before applying this proxy, we performed a simple validation to confirm that LipiORDER AUC behaves as expected as a dilution-dependent signal. Because this validation used a serial dilution series of the same EV manufacturing batch, we assumed that particle size remained unchanged across dilutions and therefore evaluated whether LipiORDER AUC alone scaled proportionally with relative EV amount (undiluted = 1.0). We prepared a serial dilution series of the same EV preparation (expressed as a relative EV amount normalized to the undiluted condition) and measured the LipiORDER AUC under the same assay settings. Across the dilution series, LipiORDER AUC increased approximately linearly with relative EV amount (Supplementary Figure S14; RSquare = 0.9994), supporting the use of LipiORDER AUC as a practical proxy for EV lipid amount under these conditions. This dilution-linearity check provides a rationale for incorporating LipiORDER AUC into the equivalent particle-count proxy used in the exploratory analyses below.

In bivariate regression, the equivalent particle-count proxy was significantly associated with potency (p = 0.0002; RSquare = 0.611939), although the association was slightly weaker than that of cGP (p < 0.0001; RSquare = 0.650721) (Supplementary Figure S15). We then tested whether this proxy explained scratch-assay potency and whether it altered the association between membrane order and potency. In a parsimonious multivariable regression including cGP and the equivalent particle-count proxy (n = 17), the overall model was significant (F (2,14) = 18.5506, p = 0.0001; RSquare = 0.726034; adjusted RSquare = 0.686896). cGP remained a significant independent predictor of PBS-normalized wound-closure potency (β = 1.1206541, p = 0.03002), whereas the equivalent particle-count proxy showed only a trend-level association (β = 0.1578746, p = 0.06998). These results indicate that the primary conclusion—membrane lipid order (cGP) best tracks potency decline across stressed conditions—remains robust even when incorporating the equivalent particle-count proxy.

## 4 Discussion

This study supports three principal findings. First, handling and storage stresses altered the EV lipid bilayer in ways that were not captured by particle size measurements, indicating that sub-lytic membrane changes (membrane loosening) can occur without obvious size shifts. Second, membrane loosening can be quantified using a polarity-sensitive membrane lipid order dye and a GP-like metric (cGP). Third, cGP captured potency decline in a keratinocyte scratch assay with higher predictive performance than conventional candidates, including protein concentration, CD63 concentration, and particle size.

Our study used EVs produced from human fibroblasts, several reports suggest that adult human fibroblasts share core phenotypic features and functional activities with mesenchymal stromal/stem cells (MSCs) and can be difficult to distinguish in some settings (Haniffa et al., 2007; Jääger and Neuman, 2011; Denu et al., 2016). These similarities support the idea that our findings could be transferable to MSC-derived EVs, particularly as a stability-indicating QC readout that detects potency deterioration within a lot. In addition, the wound-healing activity of fibroblast-derived EVs has been reported *in vivo*, and our basal-condition potency results are consistent with this prior evidence (Ahmadpour et al., 2023). However, we only tested fibroblast-derived EVs, and the same relationships should be confirmed using MSC-derived EVs and other EV sources.

EVs in this study were isolated by ultracentrifugation, which remains the most commonly used EV isolation approach in worldwide practice surveys (Royo et al., 2020; Shekari et al., 2021). Although our characterization does not fully meet all requirements of MISEV 2023 (Welsh et al., 2024), the recovered particles had a hydrodynamic diameter of approximately 135 nm and were CD63-positive by ELISA. Protein concentration was reduced compared with the original conditioned medium, and the CD63 mass per protein increased by >200-fold (Supplementary Figure S1), consistent with enrichment of CD63-bearing particles. As the present work focuses on stress-induced changes in membrane lipid order within a given preparation, we expect the direction of membrane loosening after common handling steps to be observable regardless of the purification method, although confirmatory studies using other isolation workflows (e.g., size-exclusion chromatography) are warranted. To assess whether such membrane-level changes translate into functional performance, we used a keratinocyte scratch assay as the primary potency model in this study.

We focused on wound healing as the primary EV potency context because it is one of the most extensively studied therapeutic areas for EVs (Nguyen et al., 2020; Zhu et al., 2025). Within wound-healing research, wound-closure endpoints are widely used, and scratch assays are among the most common functional readouts (Nguyen et al., 2020; Zhu et al., 2025). Therefore, we used a keratinocyte scratch assay to evaluate EV potency. We analyzed wound closure at either 24 h or 72 h depending on the experiment. Although many studies report earlier time points (often around 24 h), longer observation including 72 h has also been used in keratinocyte scratch settings (Kim et al., 2018; Glady et al., 2021; Colao et al., 2025). The apparent time-course behavior should also be interpreted in light of assay geometry. Our initial scratch gap was relatively wide (∼600 µm), whereas other published keratinocyte scratch assays may use narrower gaps such as ∼300 µm (Glady et al., 2021). Because wound closure is expressed relative to the 0 h wound area, a larger initial gap can make the percent-closure trajectory appear slower even when absolute migration rates are comparable. Accordingly, we do not interpret our data as indicating a distinct delayed biological effect of Triton-treated EVs; rather, our conclusions rely on within-experiment comparisons (stressed vs. basal EVs) under identical geometry, which remain valid.

Consistent with common practice in the field, EV dosing is often expressed as protein concentration (Kim et al., 2018; Glady et al., 2021; Shekari et al., 2021; Colao et al., 2025). In this study, however, EV dosing in the wound-healing assay was controlled by standardizing the EV production workflow (cell seeding density, conditioned-medium collection volume, and final PBS resuspension volume) and adding an equal volume fraction of EV suspension to each well (10% v/v), rather than normalizing to protein concentration. Within this standardized preparation/dosing scheme, QC parameters (e.g., particle size, protein concentration and CD63 amount) still varied between lots, and we therefore tested whether such variability could explain potency differences using regression analyses. In our datasets, conventional QC parameter such as protein concentration showed limited ability to predict potency decline, whereas corrected generalized polarization (cGP) provided stronger prediction performance. In an outlier sensitivity analysis, excluding the point shown in Figure 8D did not change the primary conclusion but revealed a tighter, directionally consistent trend in which smaller diameters tended to be associated with higher potency (Supplementary Figure S4D). This exploratory observation is consistent with a prior report that exosome size distribution can influence cellular internalization and migration-related responses, with smaller vesicles being preferentially taken up in some settings (Caponnetto et al., 2017). Because EVs are also reported to have other activities such as anti-inflammatory and anti-fibrotic effects, future studies should test whether cGP predicts potency in additional models beyond wound healing. As a reference condition for membrane disruption, we used Triton X-100, which is commonly applied as a lysis control to confirm that an observed signal depends on an intact membrane (Osteikoetxea et al., 2015; Bertokova et al., 2022). In our study, even low Triton conditions reduced scratch-assay potency while leaving the activity of soluble EGF only mildly changed. This supports the idea that small membrane defects, not only complete vesicle disruption, can be enough to reduce function and can remain difficult to detect with size measurements alone.EV stability during freezing, thawing, vortex mixing, and lyophilization is a widely recognized challenge, and reviews report that bioactivity can decrease depending on the stress type and protocol (Susa et al., 2023; Ahmadian et al., 2024). In this study, we focused on stresses likely to occur in real distribution and use: −80 °C freeze–thaw and liquid nitrogen-assisted lyophilization as storage/transport stresses, and vortex mixing as a simple pre-administration handling stress. This choice reflects near-term clinical implementation, but it also means we tested a limited set of conditions. It will also be helpful to test formulation and process changes (such as stabilizers for freezing or lyophilization) using cGP as a rapid screening readout.

Protein leakage patterns differed by stress type, but interpretation of our BCA-based readouts requires considering reagent accessibility to vesicular cargo. The BCA assay is performed under strongly alkaline conditions, under which bicinchoninic acid is expected to be largely deprotonated and anionic; therefore, in intact EVs it is likely to have limited permeability across the hydrophobic bilayer core of membrane. Consistent with this expectation, prior studies report that chemically lysing vesicular membranes can markedly increase BCA-measured “total protein”: lysis of human MSC-derived exosomes with RIPA buffer increased BCA protein by ∼3–4-fold, and lysis of bacterial outer membrane vesicles tended to yield higher BCA protein concentrations (Mosby et al., 2023; Ünal Halbutoğulları et al., 2023). Together, these observations support the view that, under unstressed conditions, intravesicular proteins are not readily detected unless membranes are disrupted or permeabilized.

In our study, increases in BCA signal after Triton exposure or logistics-relevant stresses can be interpreted as an increase in protein accessible to the assay, reflecting either (i) soluble/extravesicular proteins, (ii) proteins associated with the EV exterior (e.g., a protein corona), (iii) membrane-associated proteins with surface-exposed domains, and (iv) intravesicular proteins that become accessible when membranes are loosened or compromised. Because these possibilities cannot be distinguished by the direct readout alone, we used a 300-kDa spin filter to physically separate vesicle-sized material from soluble components and measured protein in the filtrate (flow-through) as a more selective indicator of protein present outside EVs. We adopted this filter-based readout partly inspired by dead-end ultrafiltration workflows for EV proteomics designed to reduce non-vesicular protein carryover while preserving intact vesicles (Pauwels et al., 2025). Importantly, the centrifugal filtration step imposes mechanical loading on EVs; thus, the flow-through measurement can be interpreted as capturing baseline extravesicular protein and, potentially, a stress-dependent leakage susceptibility that is revealed under centrifugal stress. Using this framework, freeze–thaw led to detectable increases without an additional centrifugation step, whereas vortex mixing and lyophilization showed limited or variable changes without separation and only modest upward shifts after centrifugal filtration. Accordingly, the data are consistent with freeze–thaw promoting leakage during the stress itself, while vortex mixing and lyophilization may primarily induce membrane weakening (increased permeability/fragility) that becomes more apparent under an external driving force, although the magnitude of the filtration-associated increase—particularly for lyophilization—was small and should be interpreted cautiously. The 300-kDa cutoff was used as a practical separation between vesicle-sized material and soluble components; it does not imply that only <300-kDa species were released, and larger macromolecules—and potentially nucleic acids—may also have leaked during membrane compromise. Importantly, we could not distinguish whether potency decline was driven mainly by loss of bioactive cargo (leakage) or by reduced cellular uptake/interaction caused by membrane loosening.

We examined whether a membrane lipid order dye could be used as a simple way to quantify membrane loosening in EVs after handling-, storage-, and reconstitution-related stresses. Polarity-sensitive membrane probes (e.g., Laurdan) and related fluorescence approaches have been used to study EV membrane lipid order and fluidity in several contexts. Some studies used such probes mainly for biophysical characterization of membrane properties rather than for potency-related evaluation (Parolini et al., 2009; Suga et al., 2021; Yasuda et al., 2022). Other studies evaluated membrane perturbation or membrane-utilizing strategies in engineering contexts, such as permeabilization for cargo loading and approaches based on protein–lipid interactions (Peruzzi et al., 2024; Auquière et al., 2025). A recent study on bovine milk EVs used Laurdan fluorescence to assess membrane integrity/stability under gastrointestinal-like conditions and noted that common physical readouts may not fully reflect functional stability (Seegobin et al., 2025). In our study, we used a membrane lipid order dye to address a different question: whether logistics-relevant stresses cause membrane loosening that is linked to potency decline. Importantly, we quantified the relationship between membrane loosening and scratch assay potency and showed that the membrane lipid order readout captured potency decline more accurately than protein concentration, CD63 concentration, or particle size. To make this readout practical and comparable across experiments, we adopted a GP-based metric and introduced a correction to reduce plate-to-plate variability: we used a corrected GP in which a plate-specific k factor derived from PBS measurements on the same plate was incorporated into the GP equation. This approach aligns with established GP methodologies using polarity-sensitive dyes (e.g., Laurdan), where a channel-correction factor (often denoted G) is applied to calibrate blue/red detection and reduce instrument-dependent bias (Brewer et al., 2010). This framework supports practical implementation and also highlights a next step for translation—defining acceptance criteria (for example, ΔcGP thresholds) that correspond to meaningful potency margins.

Notably, the membrane lipid order readout did not fully mirror the stress-specific patterns observed in the protein-leakage assays. For example, freeze–thaw showed little additional protein increase after the centrifugal filtration step, yet cGP indicated reduced membrane lipid order. This suggests that the lack of “additional” leakage under centrifugal loading does not necessarily imply preserved membrane integrity. Rather, it is plausible that freeze–thaw had already released a substantial fraction of releasable proteins during the stress itself and/or increased accessibility of intravesicular proteins in the direct BCA readout, thereby reducing the dynamic range for detecting further leakage upon subsequent centrifugation. In this view, cGP captures membrane-level perturbation even when a second-step, load-unmasked leakage signal is not clearly observed.

The mechanism by which vortex mixing, freeze–thaw, and lyophilization-reconstitution loosen EV membranes was not clarified here. EV membranes contain heterogeneous lipids, including sphingomyelin, cholesterol, and phosphatidylcholine, and their distribution into ordered raft-like domains is thought to shape membrane rigidity and function (Skotland et al., 2017; Brent et al., 2025). Membrane loosening could reflect loss or redistribution of more ordered lipids (e.g., sphingomyelin and cholesterol) and/or disruption of raft organization without an overt change in particle size. At the same time, stress responses may not reflect uniform membrane weakening across all vesicles; aggregation/clumping and/or selective survival of a more stress-resistant EV subpopulation could also contribute to the observed readouts. Future work should test this hypothesis using lipidomics (e.g., LC/MS) before and after stress to identify lipid species and domain-associated changes linked to cGP and potency.A key practical point is the difference between within-lot stability monitoring and lot-to-lot potency ranking. In our data, cGP performed best for detecting potency deterioration relative to each lot’s basal state. Bivariate analyses showed a strong association for cGP (p < 0.0001) and nominal or borderline associations for protein (p = 0.0505) and CD63 (p = 0.0408). However, in the multivariable regression, these latter associations were no longer significant, and only cGP remained significant after adjustment, supporting cGP as the most robust independent predictor of scratch-assay potency in this model. Consistent with this, in an exploratory sensitivity analysis we fit a parsimonious model including cGP and an equivalent particle-count proxy derived from LipiORDER AUC and DLS-derived particle size, given the modest sample size and the risk of overfitting in multivariable regression (Babyak et al., 2004). As a methodological note, because this proxy uses the DLS hydrodynamic diameter (Z-average) for surface-area normalization, it can be disproportionately influenced by larger, highly scattering species in heterogeneous or partially aggregated samples. Accordingly, the proxy should not be interpreted as an absolute measure of EV particle number, but rather as a relative, exploratory index intended to provide supportive context in the absence of direct particle-count instrumentation. With these interpretive considerations in mind, cGP remained independently associated with potency, whereas the proxy showed only a trend-level contribution. By contrast, baseline potency differences between repeatedly manufactured lots produced from the same fibroblast donor under an identical standardized workflow (EV01–EV06) were not explained well by membrane lipid order alone, which is reasonable because intrinsic potency likely depends on multiple biological factors beyond membrane packing. Therefore, we view membrane lipid order as a complementary QC tool that is most useful for stability assessment after handling, storage, or reconstitution.

From a practical perspective, checking cGP after storage or after vortex mixing could provide a simple way to confirm stability and to support go/no-go decisions before administration. Because the assay requires only a membrane lipid order dye and a standard plate reader, it may be useful across development, distribution, and clinical handling as a rapid, potency-relevant screen for membrane compromise. Recent position papers and guidance on EV-based medicinal products emphasize that potency assays are needed, but they can be time-consuming and variable, especially when they rely on cell responses. Our results suggest a practical role for membrane lipid order as a stability-indicating attribute: it can help answer a simple QC question after transport or storage—whether a given lot has lost potency compared with its own baseline.

In summary, a membrane lipid order–based readout provides a simple and rapid way to detect potency decline after handling, storage, and reconstitution stresses. It can complement conventional characterization by capturing membrane loosening that may otherwise go unnoticed.

## 5 Conclusion

A fluorescence-based membrane lipid order index (cGP) measured using a polarity-sensitive membrane lipid order dye sensitively tracked potency deterioration of therapeutic EVs under multiple handling and storage stress conditions. While not sufficient to explain baseline lot-to-lot potency, membrane lipid order offers a practical stability-oriented QC metric to complement conventional EV characterization and to support distribution and storage control.

## Data Availability

The original contributions presented in the study are included in the article/Supplementary Material, further inquiries can be directed to the corresponding author.
