# Supplementary material for: Membrane lipid order predicts potency decline in therapeutic extracellular vesicles following handling, storage, and reconstitution stress
Source: Front Bioeng Biotechnol. 2026 Apr 13;14:1795387. doi: 10.3389/fbioe.2026.1795387 (PMC13111347; doi:10.3389/fbioe.2026.1795387)
Supplement: Supplementary file 1 [file Supplementaryfile1.docx]

Supplementary Material

**Membrane Lipid Order Predicts Potency Decline in Therapeutic Extracellular Vesicles Following Handling, Storage, and Reconstitution Stress**

Kenichi Tamura 1, Shinji Takeoka 1,2

1Cooperative Major in Advanced Biomedical Sciences of Tokyo Women’s Medical University and Waseda University, 2-2, TWIns, Wakamatsu-cho, Shinjuku-ku, Tokyo 162-8480 JAPAN

2Waseda Research Institute for Science and Engineering, 3-4-1 Okubo, Shinjuku-ku, Tokyo 169-8555 JAPAN

* Correspondence:

Shinji Takeoka, 813-5369-7324, [takeoka@waseda.jp](mailto:takeoka@waseda.jp)

## Supplementary Figures


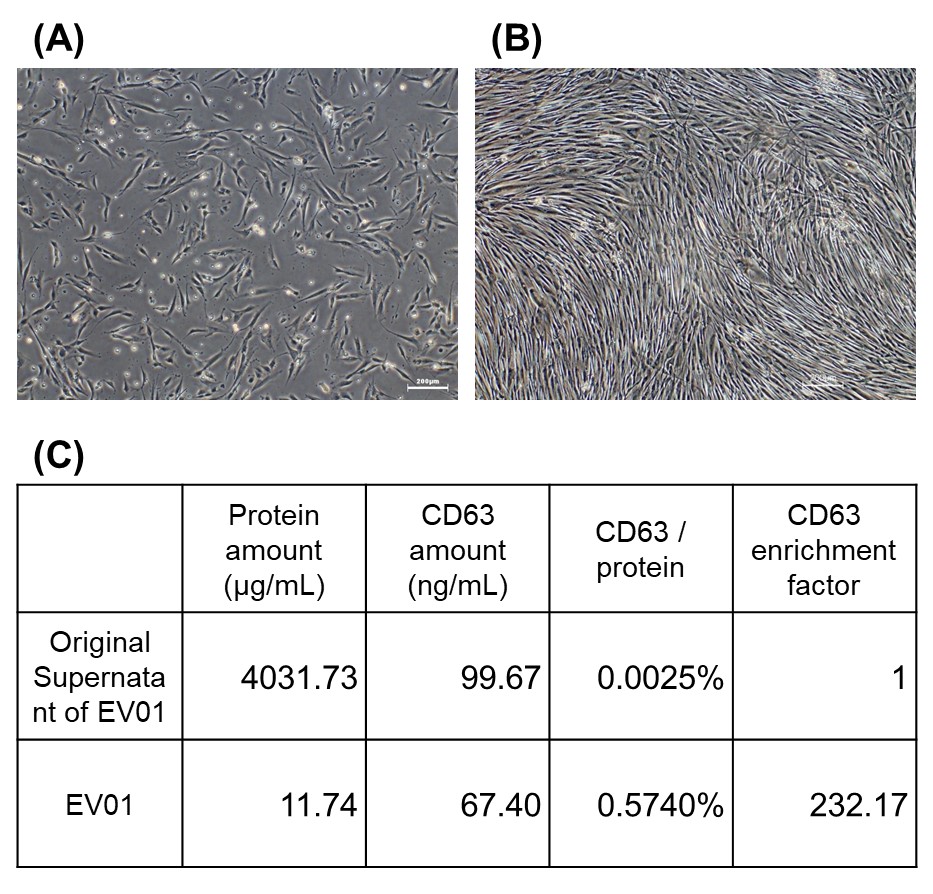


**Supplementary Figure 1.** Representative phase-contrast images (4×) of human fibroblasts are shown. (A): proliferating fibroblasts under routine culture conditions. (B): fibroblasts seeded at 10,000 cells/cm^2 for EV production/collection, showing the typical morphology at the time of conditioned-medium harvest. (C): CD63 content normalized to total protein (CD63 per unit protein mass) in the conditioned medium (pre-purification) and in the purified EV preparation, demonstrating enrichment of the EV marker CD63 after EV isolation. Scale bars: 200 µm.


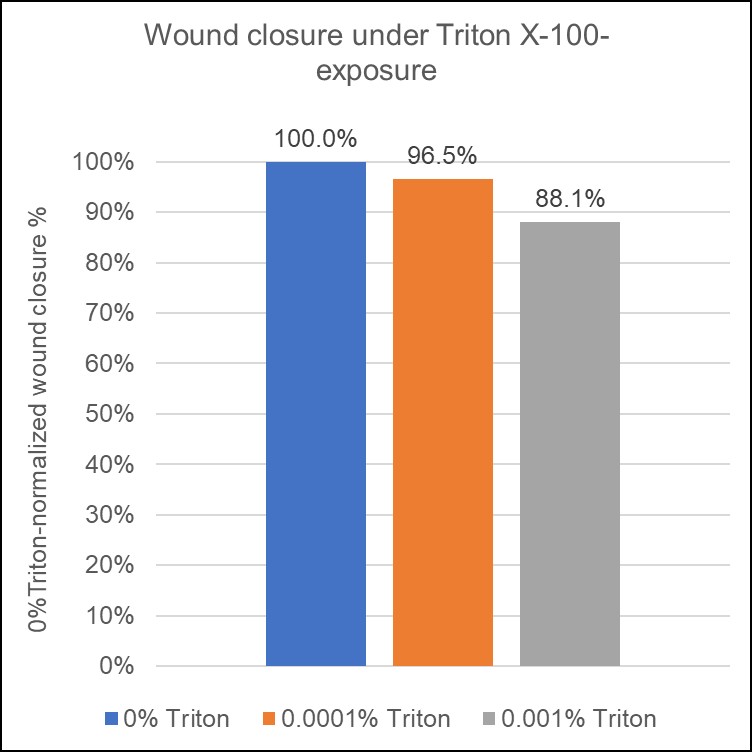


**Supplementary Figure 2**. Keratinocyte scratch-wound closure under Triton X-100 exposure. Immortalized human keratinocytes were exposed to Triton X-100 at 0%, 0.0001%, or 0.001% (v/v), and scratch-wound closure was quantified at the assay endpoint. Wound closure is shown as the percentage normalized to the 0% Triton condition (set to 100%) within the same experiment. Data are from duplicate wells in one experiment.


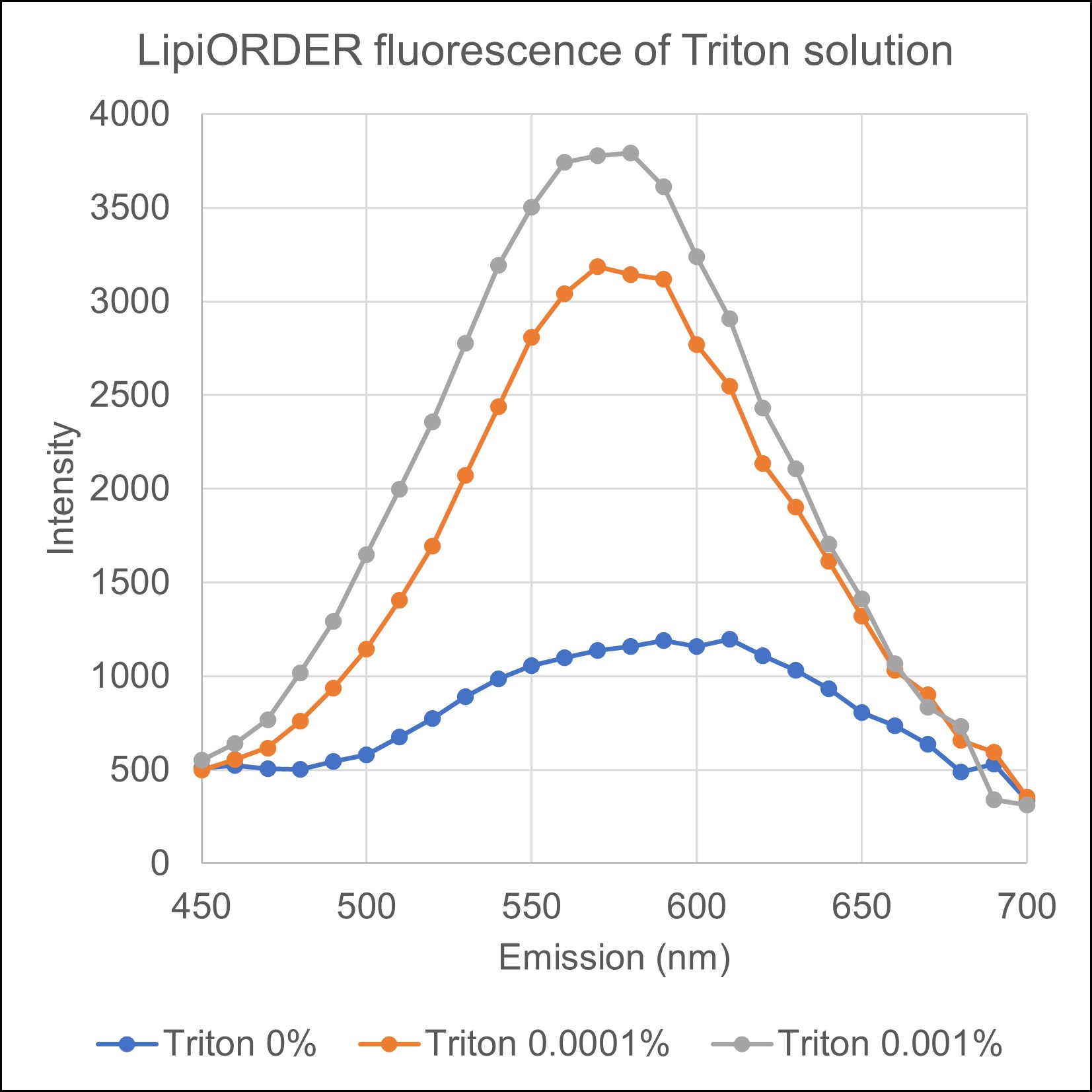


**Supplementary Figure 3.** LipiORDER fluorescence emission spectra were recorded for Triton X-100 solutions at 0%, 0.0001%, and 0.001% (v/v) in the absence of EVs. Data are from triplicate wells in one experiment.


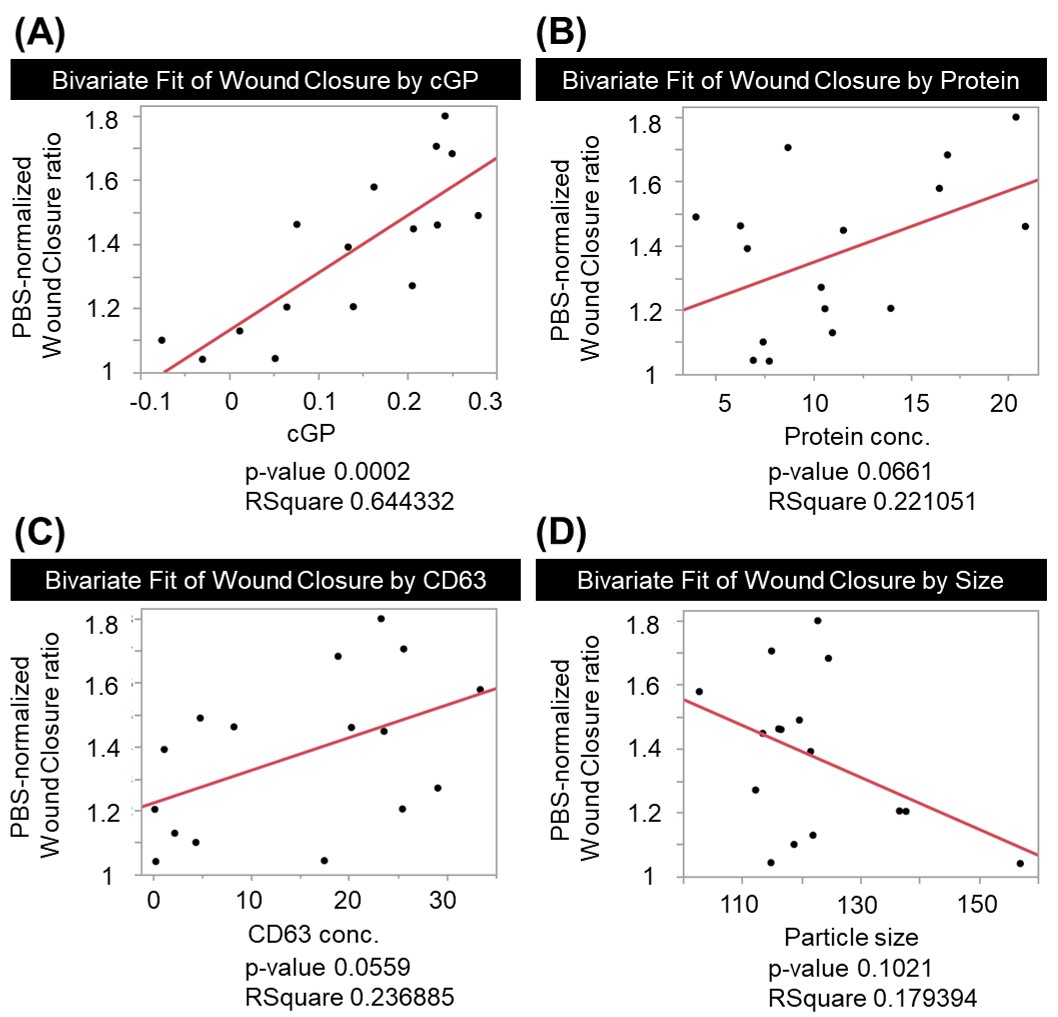


**Supplementary Figure 4.** Sensitivity analysis of bivariate associations between potency and QC candidates after excluding the particle-size outlier (>350 nm). Scratch-assay potency is expressed as the ratio of wound-closure (%) relative to the matched PBS vehicle control in the same experiment. Predictor variables are (A): cGP, (B): protein (µg/mL), (C): CD63 (ng/mL), and (D): particle size (nm). Red lines indicate fitted linear regression trends for each bivariate model.


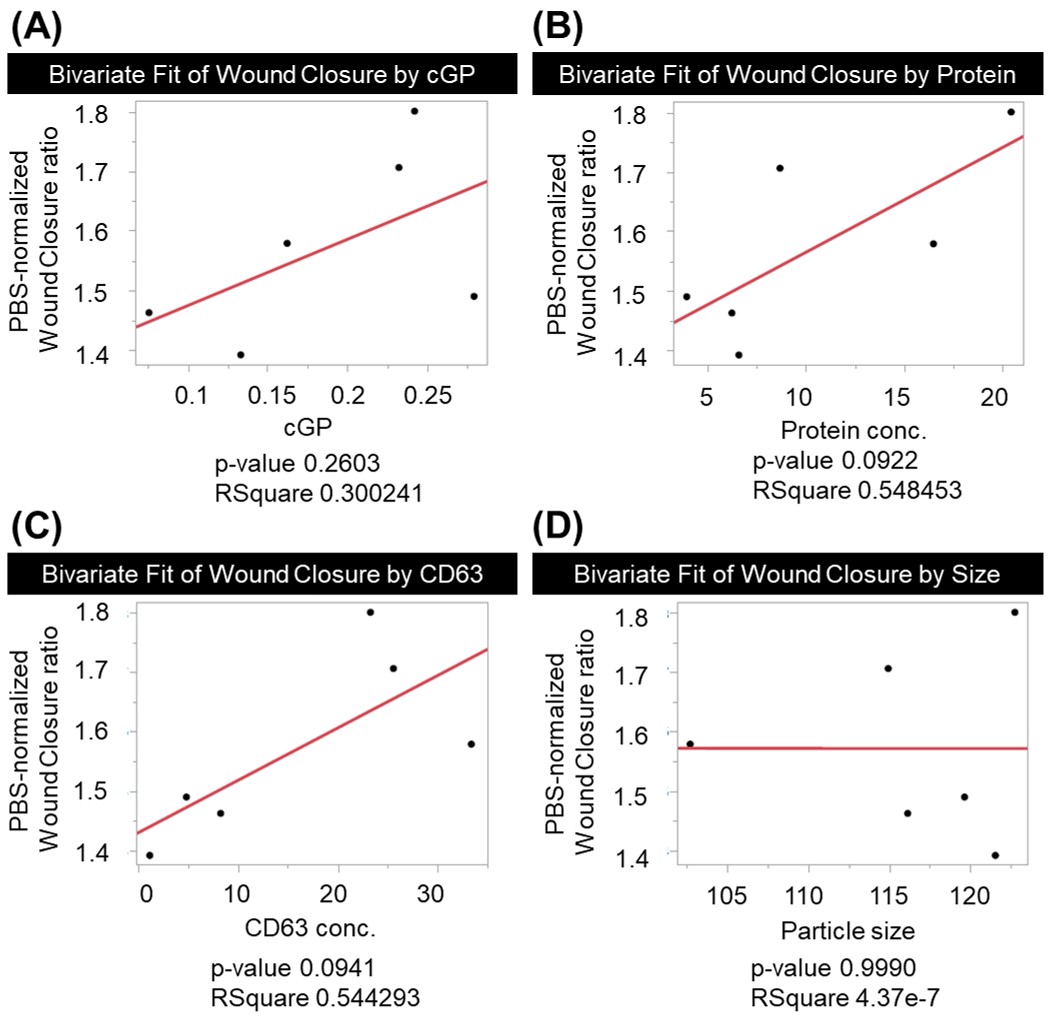


**Supplementary Figure 5.** Stratified bivariate analyses of basal lot-to-lot potency. Bivariate relationships between keratinocyte scratch-assay potency under basal conditions (y-axis) and candidate QC measures across independently manufactured EV lots without handling/storage stresses (x-axes). Scratch-assay potency is expressed as the ratio of wound-closure (%) relative to the matched PBS vehicle control in the same experiment. Predictor variables are (A): cGP, (B): protein (µg/mL), (C): CD63 (ng/mL), and (D): particle size (nm). Red lines indicate fitted linear regression trends for each bivariate model.


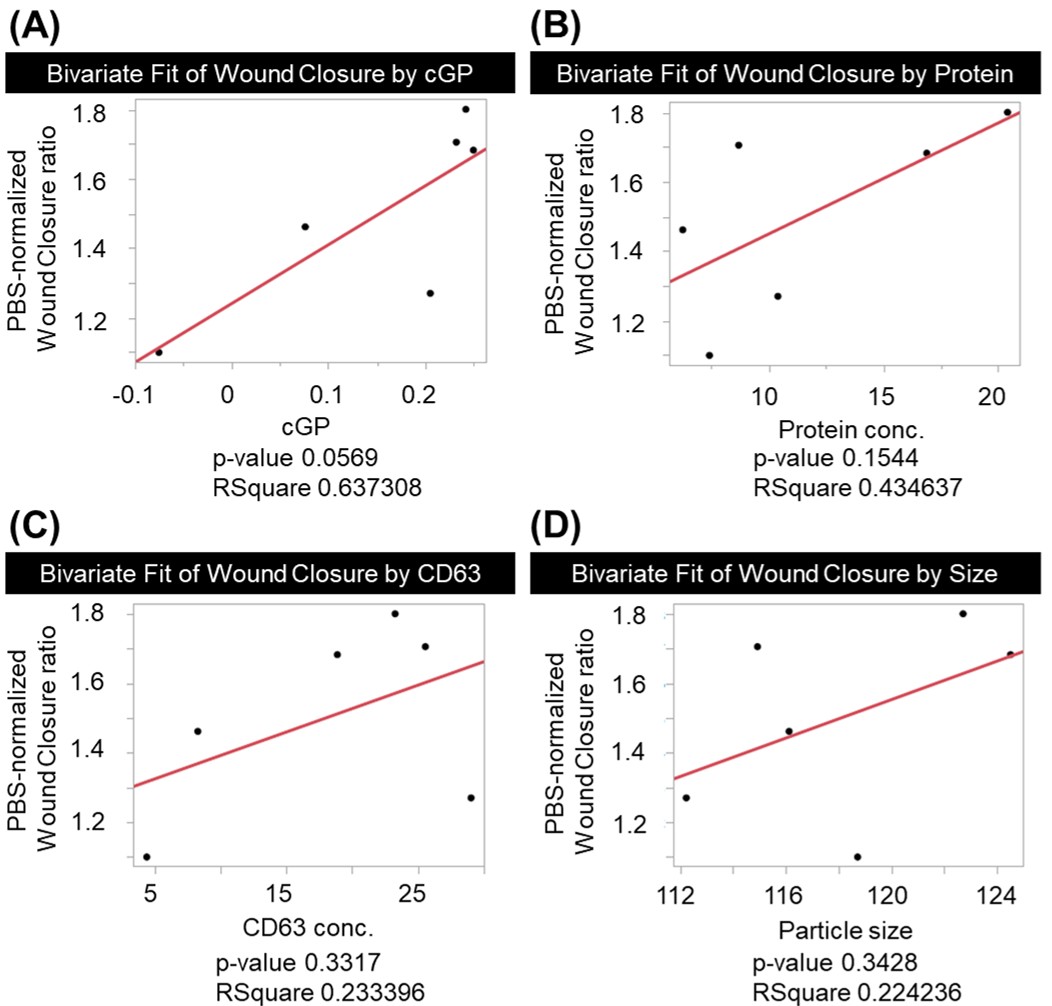


**Supplementary Figure 6.** Stratified bivariate analyses of potency versus QC candidates after vortex mixing. Scatter plots show PBS-normalized wound-closure potency in the keratinocyte scratch assay versus (A): cGP, (B): protein (µg/mL), (C): CD63 (ng/mL), and (D): particle size (nm) for vortex-mixed and untreated EV samples. Red lines indicate least-squares regression fits; p-values and RSquares are reported in each panel.


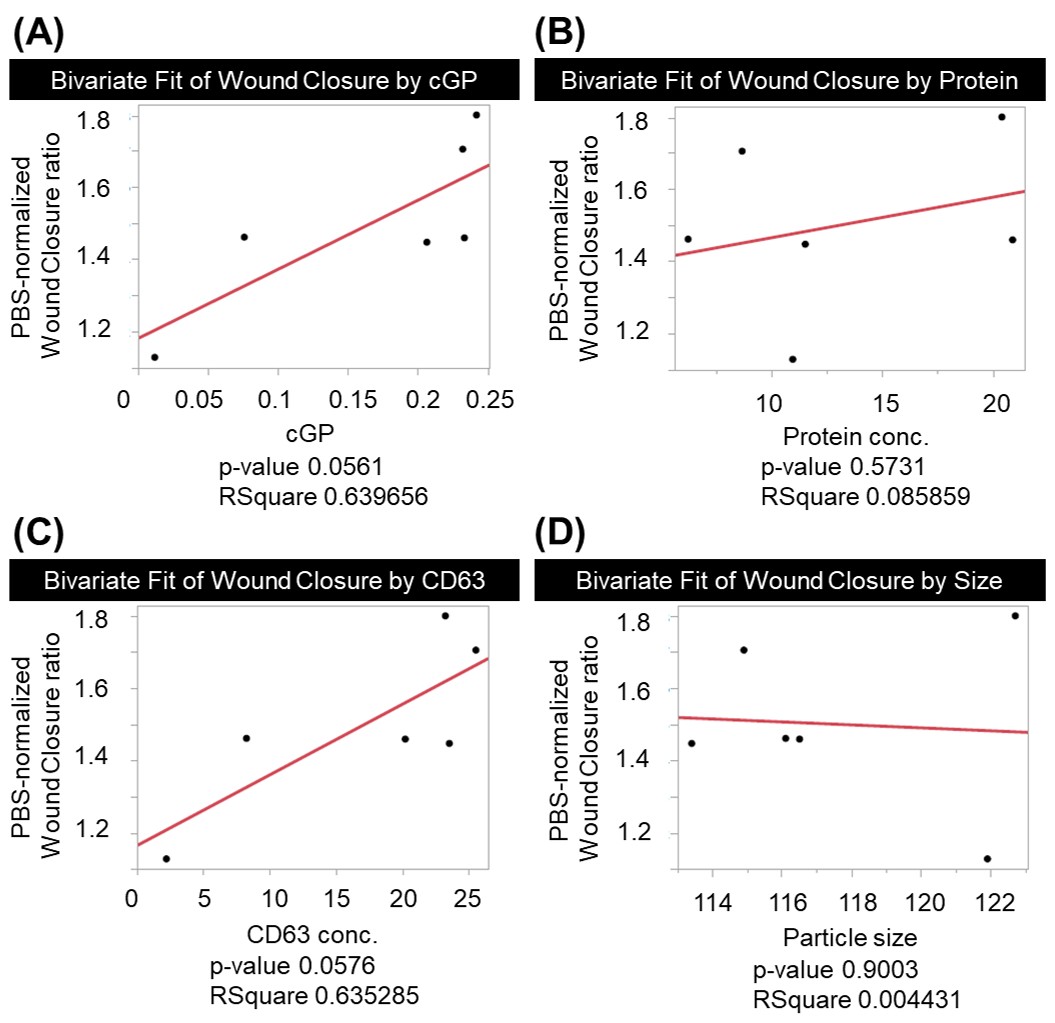


**Supplementary Figure 7.** Stratified bivariate analyses of potency versus QC candidates after freeze-thaw cycling. Scatter plots show PBS-normalized wound-closure potency in the keratinocyte scratch assay versus (A): cGP, (B): protein (µg/mL), (C): CD63 (ng/mL), and (D): particle size (nm) for freeze-thawed and untreated EV samples. Red lines indicate least-squares regression fits; p-values and RSquares are reported in each panel.


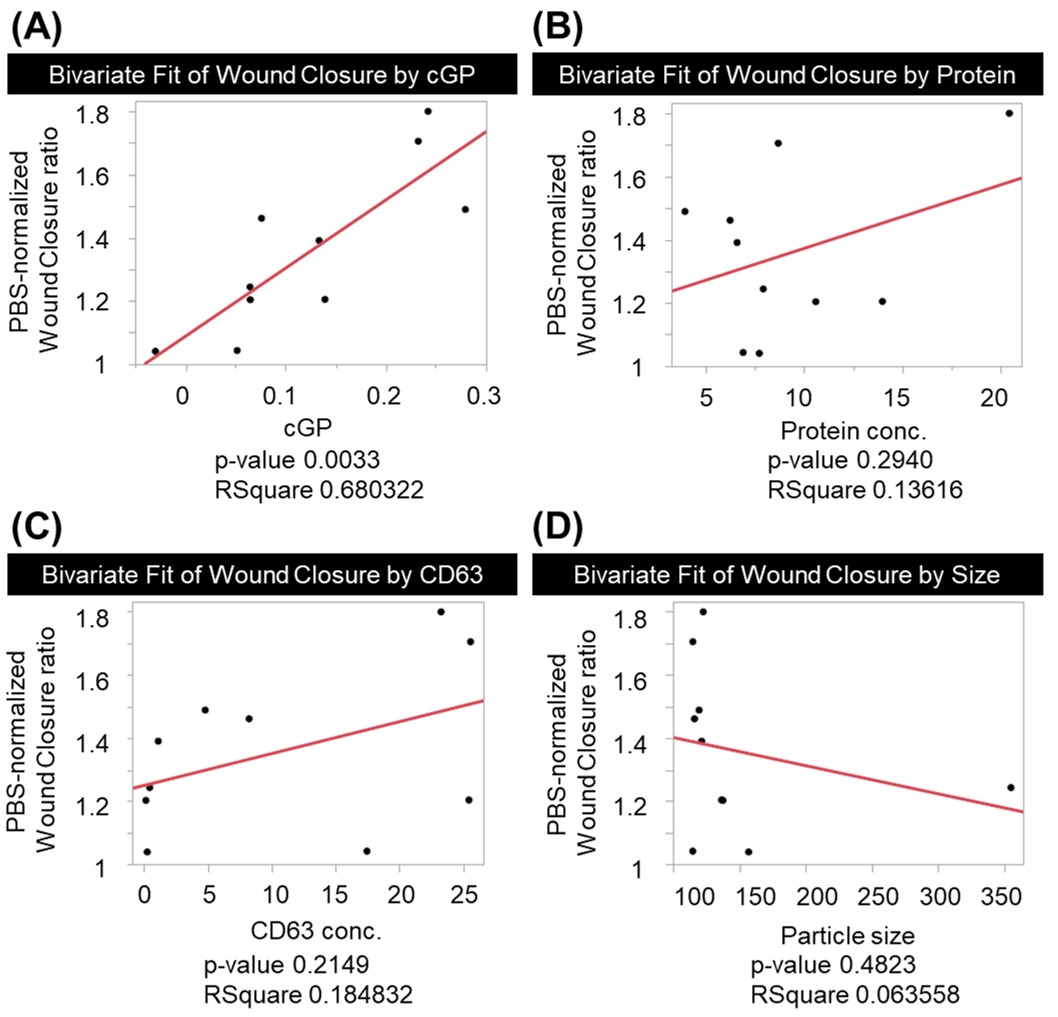


**Supplementary Figure 8.** Stratified bivariate analyses of potency versus QC candidates after lyophilization-reconstitution. Scatter plots show PBS-normalized wound-closure potency in the keratinocyte scratch assay versus (A): cGP, (B): protein (µg/mL), (C): CD63 (ng/mL), and (D): particle size (nm) for lyophilized-reconstituted and untreated EV samples. Red lines indicate least-squares regression fits; p-values and RSquares are reported in each panel.


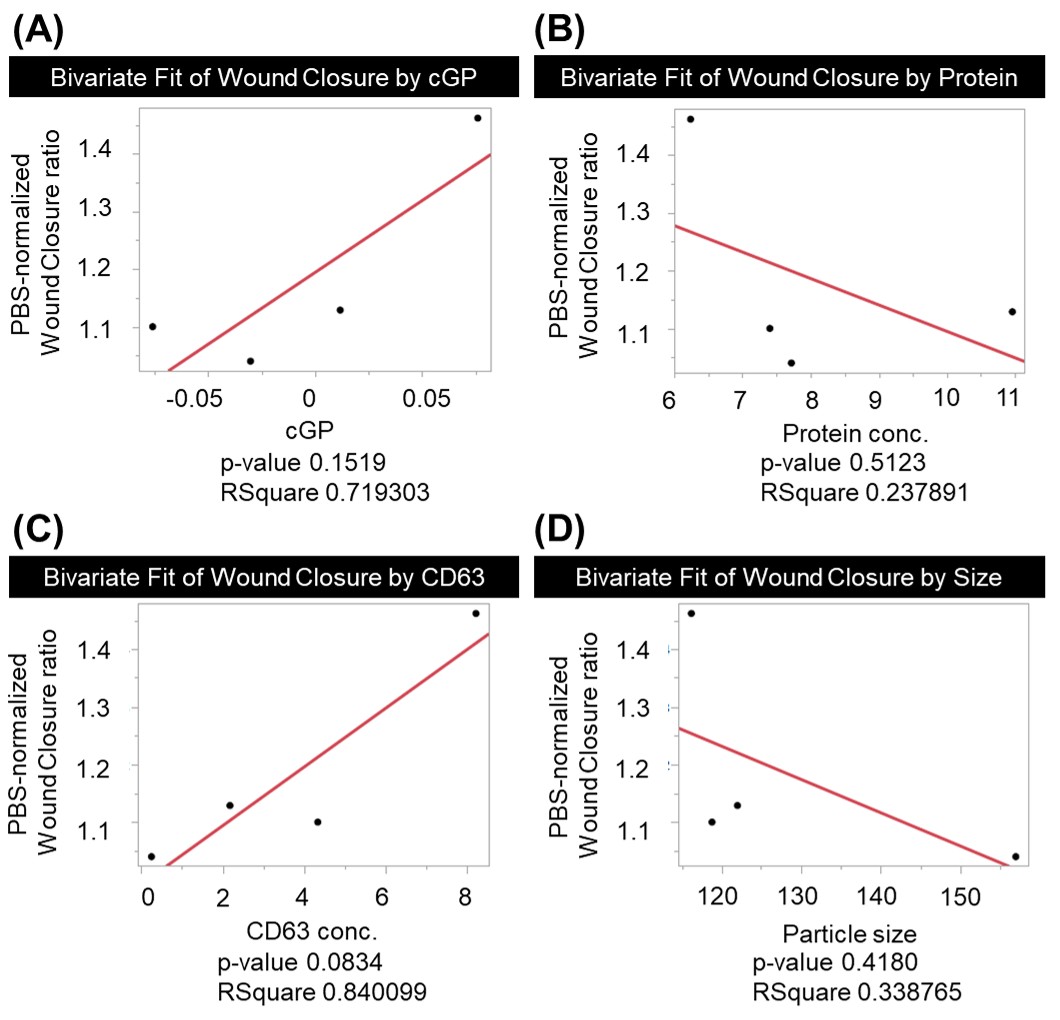


**Supplementary Figure 9.** Lot EV01: associations between scratch-assay potency and QC candidates. Using EV01, bivariate linear regression was performed between keratinocyte scratch-assay potency (expressed as the wound-closure ratio relative to the matched PBS vehicle control) and each candidate QC measure ((A): cGP, (B): protein (µg/mL), (C): CD63 (ng/mL), and (D): particle size (nm)) for handling/storage stressed and untreated EV samples. Each panel reports the p-value and RSquare for each lot.


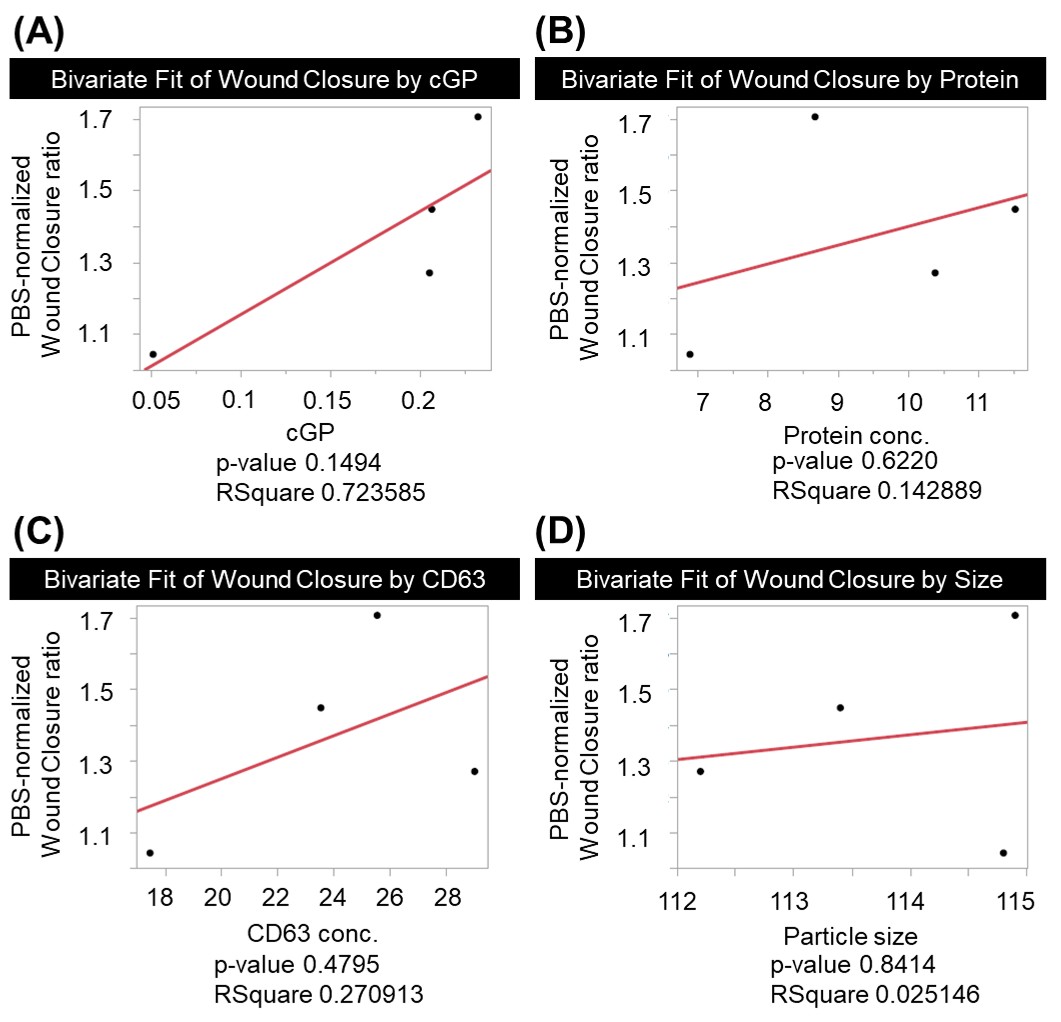


**Supplementary Figure 10.** Lot EV03: associations between scratch-assay potency and QC candidates. Using EV03, bivariate linear regression was performed between keratinocyte scratch-assay potency (expressed as the wound-closure ratio relative to the matched PBS vehicle control) and each candidate QC measure ((A): cGP, (B): protein (µg/mL), (C): CD63 (ng/mL), and (D): particle size (nm)) for handling/storage stressed and untreated EV samples. Each panel reports the p-value and RSquare for each lot.


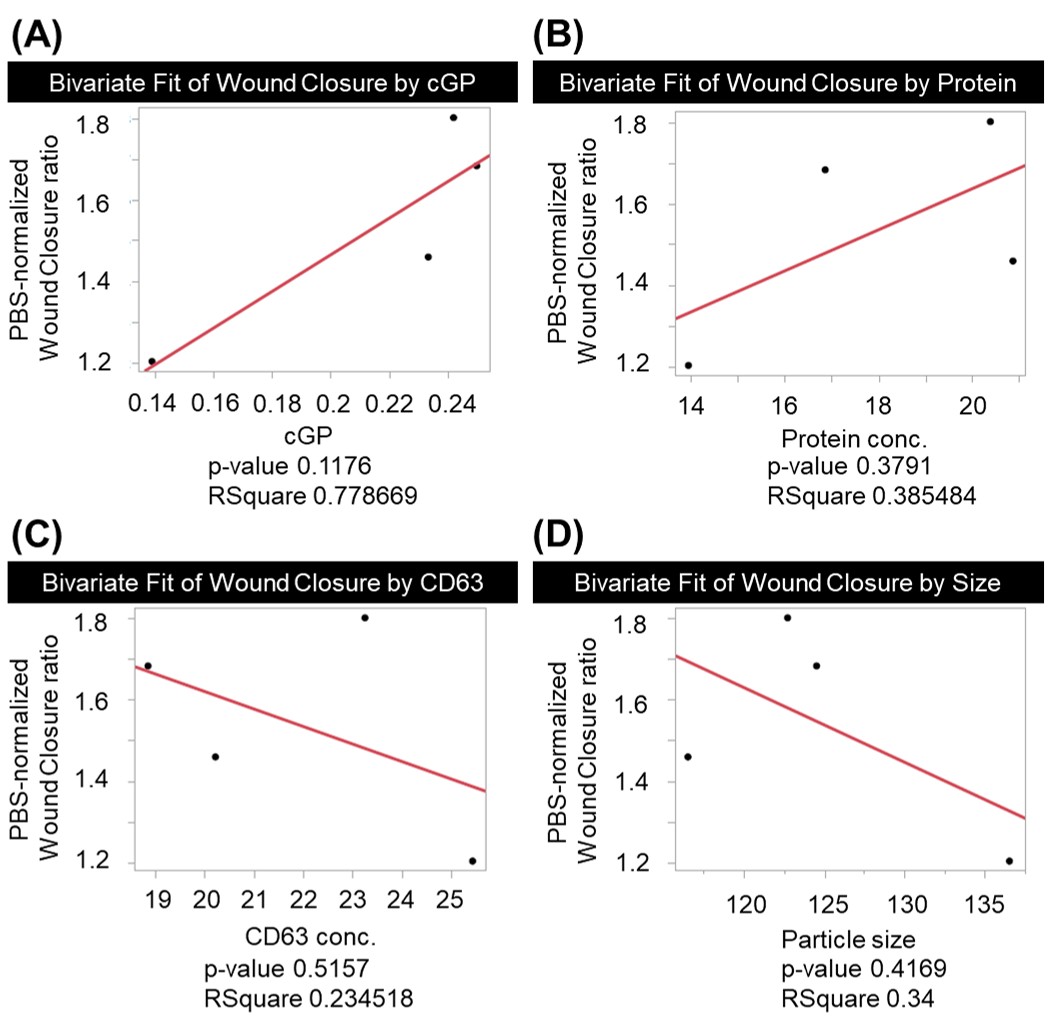


**Supplementary Figure 11.** Lot EV04: associations between scratch-assay potency and QC candidates. Using EV04, bivariate linear regression was performed between keratinocyte scratch-assay potency (expressed as the wound-closure ratio relative to the matched PBS vehicle control) and each candidate QC measure ((A): cGP, (B): protein (µg/mL), (C): CD63 (ng/mL), and (D): particle size (nm)) for handling/storage stressed and untreated EV samples. Each panel reports the p-value and RSquare for each lot.


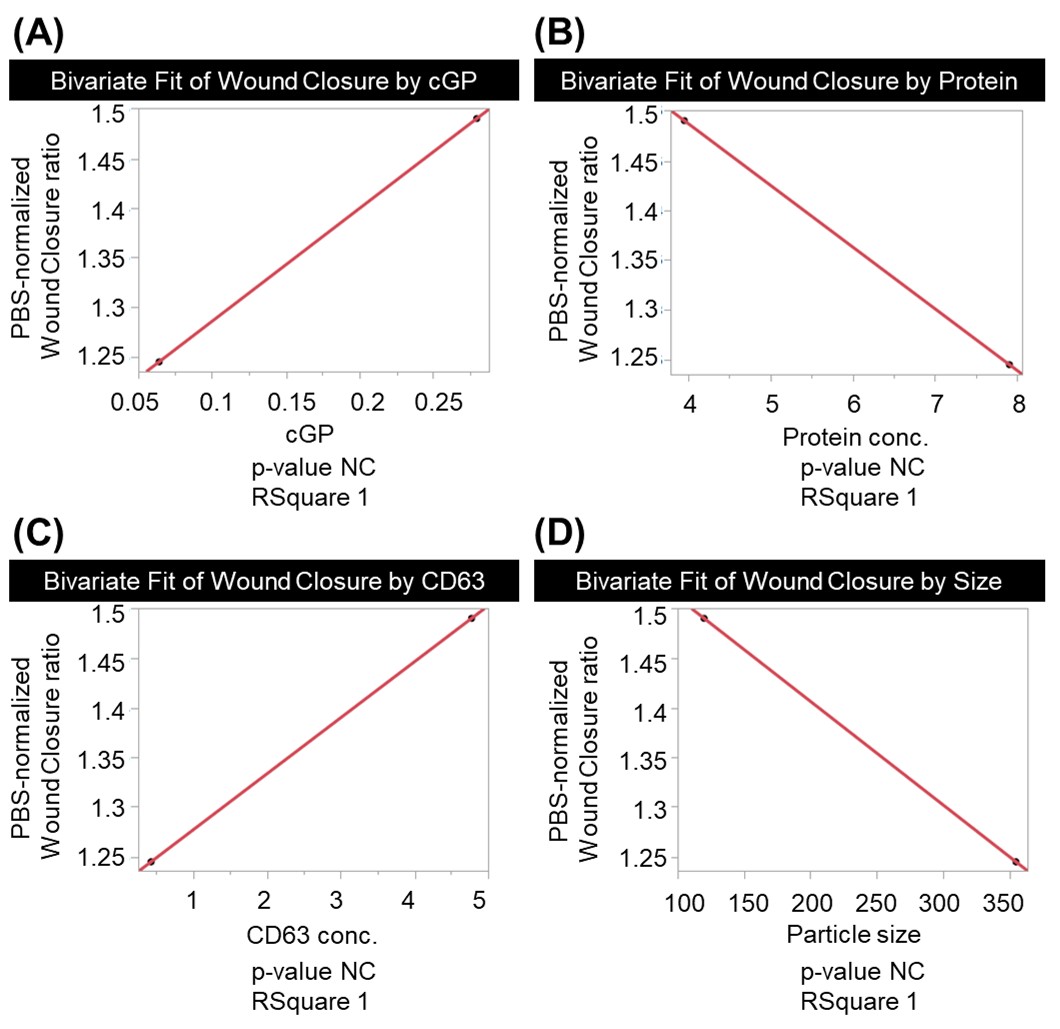


**Supplementary Figure 12.** Lot EV05: associations between scratch-assay potency and QC candidates. Using EV05, bivariate linear regression was performed between keratinocyte scratch-assay potency (expressed as the wound-closure ratio relative to the matched PBS vehicle control) and each candidate QC measure ((A): cGP, (B): protein (µg/mL), (C): CD63 (ng/mL), and (D): particle size (nm)) for handling/storage stressed and untreated EV samples. Each panel reports the p-value and RSquare for each lot. NC: non-calculable.


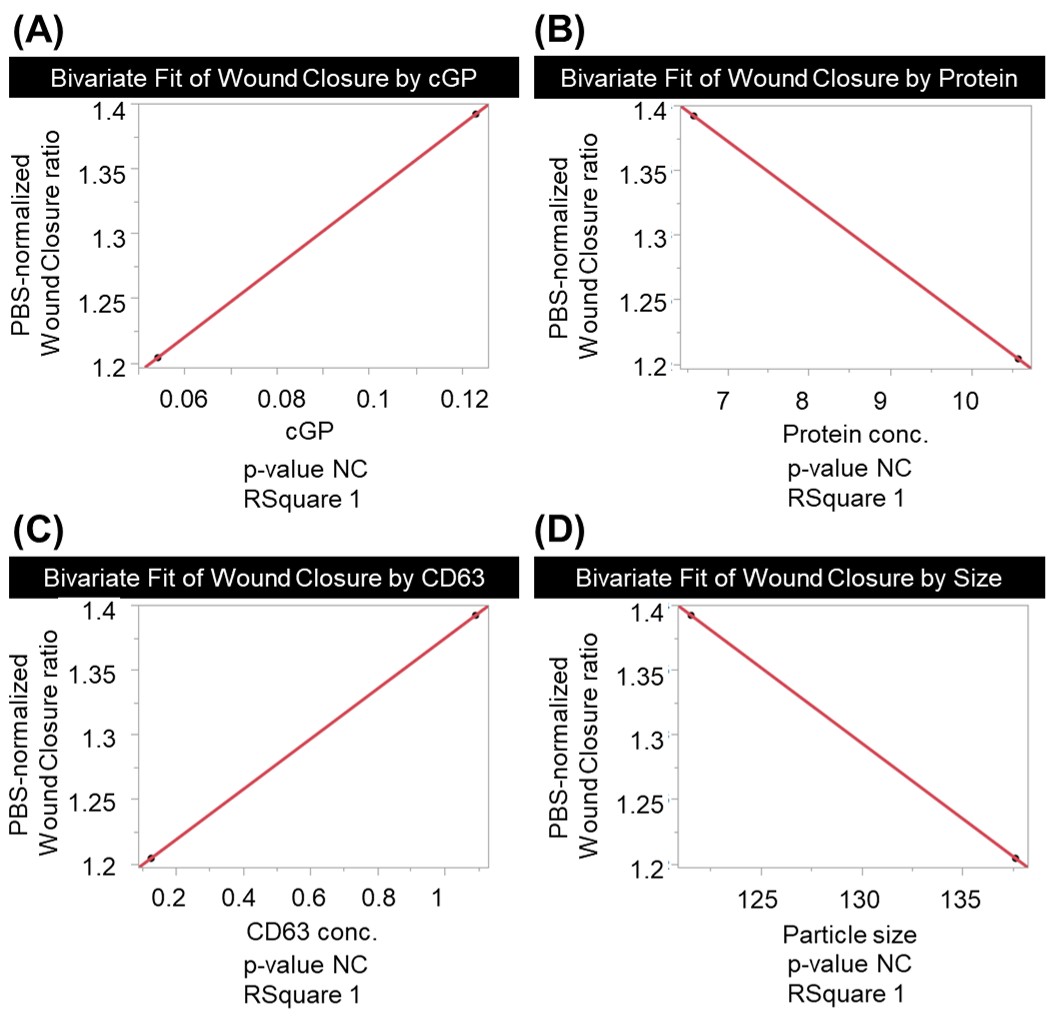


**Supplementary Figure 13.** Lot EV06: associations between scratch-assay potency and QC candidates. Using EV06, bivariate linear regression was performed between keratinocyte scratch-assay potency (expressed as the wound-closure ratio relative to the matched PBS vehicle control) and each candidate QC measure ((A): cGP, (B): protein (µg/mL), (C): CD63 (ng/mL), and (D): particle size (nm)) for handling/storage stressed and untreated EV samples. Each panel reports the p-value and RSquare for each lot. NC: non-calculable.


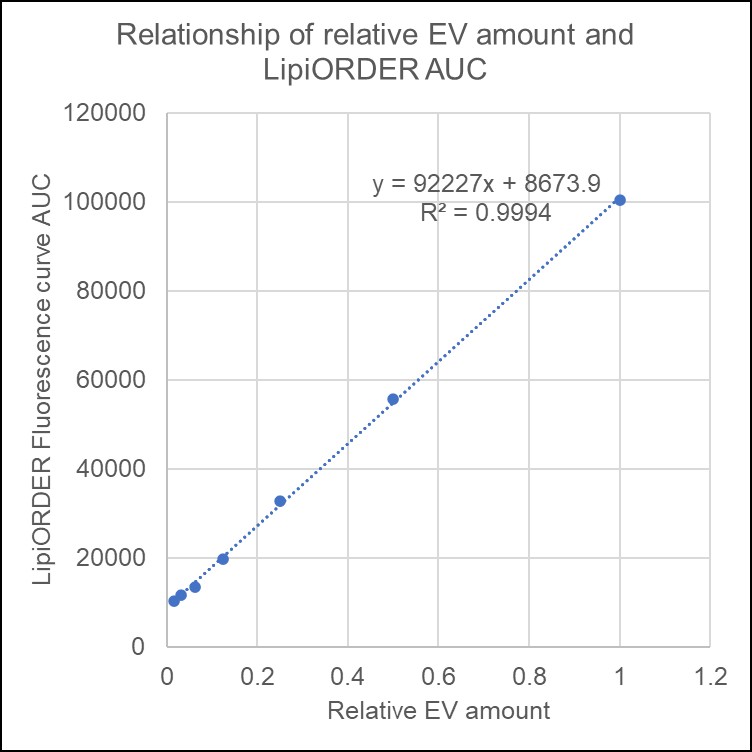


**Supplementary Figure 14.** Linearity of LipiORDER fluorescence area-under-the-curve (AUC) across a serial dilution series of an EV preparation. The dotted line indicates the fitted linear regression; the regression equation and RSquare are shown in the panel. Data are from triplicate wells in one experiment.


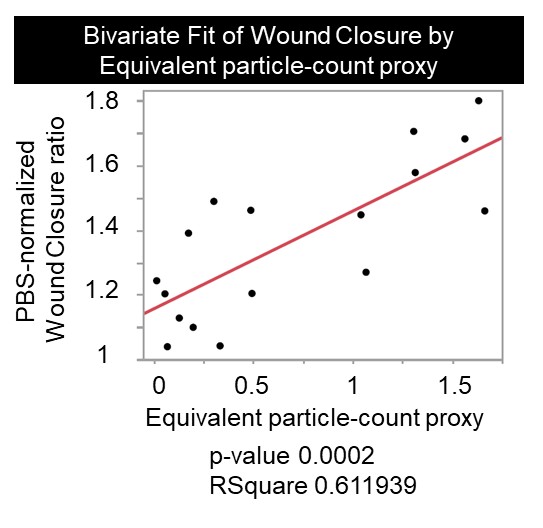


**Supplementary Figure 15.** Exploratory analysis of bivariate associations between potency and equivalent particle-count proxy. Scratch-assay potency is expressed as the ratio of wound-closure (%) relative to the matched PBS vehicle control in the same experiment. Red lines indicate fitted linear regression trends for each bivariate model.
